# Extracellular vesicles and vesicle-free secretome of the protozoa *Acanthamoeba castellanii* under homeostasis and nutritional stress and their damaging potential to host cells

**DOI:** 10.1080/21505594.2018.1451184

**Published:** 2018-05-04

**Authors:** Diego de Souza Gonçalves, Marina da Silva Ferreira, Susie Coutinho Liedke, Kamilla Xavier Gomes, Gabriel Afonso de Oliveira, Pedro Ernesto Lopes Leão, Gabriele Vargas Cesar, Sergio H. Seabra, Juliana Reis Cortines, Arturo Casadevall, Leonardo Nimrichter, Gilberto Barbosa Domont, Magno Rodrigues Junqueira, Jose Mauro Peralta, Allan J. Guimaraes

**Affiliations:** a Departamento de Microbiologia e Parasitologia, Instituto Biomédico, Universidade Federal Fluminense, Niterói, Brazil; b Departamento de Imunologia, Instituto de Microbiologia Professor Paulo de Góes, Universidade Federal do Rio de Janeiro, Rio de Janeiro, Brazil; c Laboratório de Glicobiologia de Eucariotos, Instituto de Microbiologia Paulo de Góes, Universidade Federal do Rio de Janeiro, Rio de Janeiro, Brazil; d Laboratório de Tecnologia em Cultura de Células, Centro Universitário Estadual da Zona Oeste (UEZO), Rio de Janeiro, Brazil; e Departamento de Virologia, Instituto de Microbiologia Professor Paulo de Góes, Universidade Federal do Rio de Janeiro, Rio de Janeiro, Brazil; f Department of Molecular Microbiology and Immunology, Johns Hopkins Bloomberg School of Public Health, Baltimore, MD, USA; g Departamento de Bioquímica, Instituto de Química, Universidade Federal do Rio de Janeiro, Rio de Janeiro, Brazil

**Keywords:** *Acanthamoeba castellanii*, exosomes, pathogenesis, secretome, virulence factors

## Abstract

*Acanthamoeba castellanii* (*Ac*) are ubiquitously distributed in nature, and by contaminating medical devices such as heart valves and contact lenses, they cause a broad range of clinical presentations to humans. Although several molecules have been described to play a role in Ac pathogenesis, including parasite host-tissue invasion and escaping of host-defense, little information is available on their mechanisms of secretion. Herein, we describe the molecular components secreted by Ac, under different protein availability conditions to simulate host niches. *Ac* extracellular vesicles (EVs) were morphologically and biochemically characterized. Dynamic light scattering analysis of *Ac* EVs identified polydisperse populations, which correlated to electron microscopy measurements. High-performance thin liquid chromatography of *Ac* EVs identified phospholipids, steryl-esters, sterol and free-fatty acid, the last two also characterized by GC-MS. Secretome composition (EVs and EVs-free supernatants) was also determined and proteins biological functions classified. In peptone-yeast-glucose (PYG) medium, a total of 179 proteins were identified (21 common proteins, 89 exclusive of EVs and 69 in EVs-free supernatant). In glucose alone, 205 proteins were identified (134 in EVs, 14 common and 57 proteins in EVs-free supernatant). From those, stress response, oxidative and protein and amino acid metabolism proteins prevailed. Qualitative differences were observed on carbohydrate metabolism enzymes from Krebs cycle and pentose phosphate shunt. Serine proteases and metalloproteinases predominated. Analysis of the cytotoxicity of Ac EVs (upon uptake) and EVs-free supernatant to epithelial and glioblastoma cells revealed a dose-dependent effect. Therefore, the Ac secretome differs depending on nutrient conditions, and is also likely to vary during infection.

## Introduction

Protozoa from the genus *Acanthamoeba* have been isolated from distinct environments around the world, such as soil, water reservoirs, public toilets or environmental water sources, such as lakes and rivers [1,2]. Medical devices, i.e., heart valves [3] and contact lenses, are also platforms for the growth of *Acanthamoeba* sp.; the last mainly by the usage of contaminated cleaning solutions [4–6].

The *Acanthamoeba* genus is comprised of single-celled microorganisms, which features two distinct phases of life cycle: the free-living and metabolically active stage trophozoites and the resistant phase stage called cyst. The parasitic trophozoites are motile and display spiny-like projections on their surfaces, called acanthapodia. These movements are important for *Acanthamoeba* nutrient acquisition, which happens essentially by phagocytosis of bacteria or yeasts. On the other hand, the non-motile cyst form has a double-layered wall, which provides resistance to environmental adversities such as extremes of pH, high temperatures and antimicrobials [6–9].

*Acanthamoeba* sp. infections are associated with a broad range of clinical presentations in humans, but the most common conditions involve the eye and central nervous system infections [6,10–12]. *Acanthamoeba* keratitis is one of the most common and serious clinical manifestations caused by this organism, followed by encephalitis; both can cause irreversible damage if not treated properly [10,11,13–15].

Several molecules have been described to have a role on *Acanthamoeba* pathogenesis, helping the protozoa to invade the host tissue and escape their defense mechanisms [16,17]. The secretion of glycoproteins, proteases and others molecules was correlated with the pathogenic potential of these organisms [18]. Among these components, proteases have been documented as the major virulence factors and could mediate host tissue destruction and digestion of phagocytosed particles within the microorganism [19–21]. In addition to the effects of proteases such as elastases, metalloproteases, serine and cysteine proteases regarding tissue invasion [21–26], they are also directly related to mannose-binding proteins mediated adhesion, playing important role in the interaction of *Acanthamoeba* and the host cell.

In recent years, extracellular vesicles (EVs) have been described as secretion mechanisms for a wide range of molecules to reach the extracellular environment in a large number of organisms [27–31]. The cellular origin of these vesicular molecules and the exact mechanism by which they are produced are yet to be determined, although numerous studies suggest that this process involves components of both conventional and non-conventional secretion pathways [32–34]. However, morphological and biochemical characteristics indicate that they are similar to those described in several mammalian cells [35], containing lipids, nucleic acids, protein and polysaccharide components [31,36–38]. In the case of important human fungal pathogens, many of those components are associated with virulence [33,36,37,39,40].

The production of EVs have also been described in such protozoa as *Plasmodium* sp [41]., *Leishmania donovani* and *L. major* [42,43], *Trypanosoma brucei* [44,45], *T. cruzii* [46,47], in helmints such as the nematodes *Echinostoma caproni* and *Fasciola hepatica* [48], *Helingmosomoides polygrus* [49], *Teladorsagia circumcincta* [50], the trematode *Dicrocoelium dendriticum* [51] and the amoeba *Dictyostelium discoideum* [52]. Some reports have stated the importance of these exosomes during host-parasite interaction, working as communication mediators between the different cells from distinct organisms, therefore bearing a potential therapeutic target for infectious diseases [40,41].

The secretory mechanisms used by amoebas are still unclear, but there is a parallel to the release of exosomes described in eukaryotes such as parasites, fungi and mammals [53–56]. In these models, EVs have been described to be involved in the secretion of proteinases into the extracellular environment, with important implications for the pathogenesis and virulence [18,40,41,57]; EVs from *Acanthamoeba* sp. may contain important virulence factors which, when secreted, could also damage host tissues. Our goal in this study was to characterize the EVs produced and secreted by *A. castellanii*. As this organism can be found, either within the host or in the environment, in several locations with the most diverse protein concentrations, we characterized the secretome profile of *A. castellanii*, including the isolation EVs and EV-free fractions, in conditions of distinct availability of protein sources. As EVs were taken up by host cells, the damaging potential and lethal activity of EVs and EVs-free supernatant to host cells were evaluated. Our results suggest the participation of the secretome of *A. castellanii* on pathogenesis, which could provide a new option for the therapeutic interventions of *A. castellanii* infections.

## Results

### A. castellanii trophozoites shed membrane vesicles

**Figure 1.**
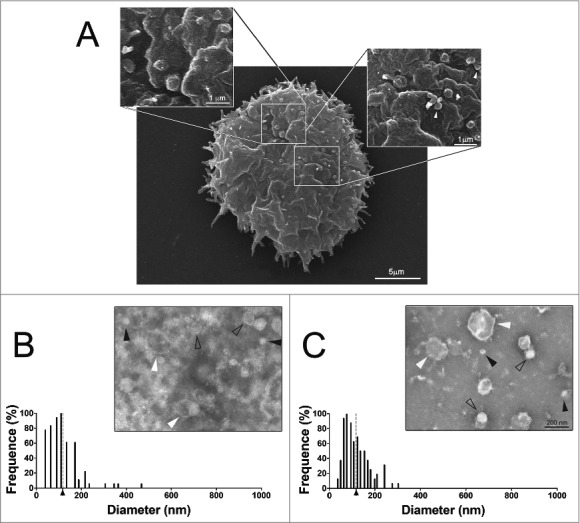
Characterization of EVs secreted by *A. castellanii* (A) Scanning microscopy of *A. castellanii* demonstrating the ultrastructure and topography of the amoeba. At the cell´s surface, it is possible to visualize the vesicle shedding, with evagination from the membrane of sphere-like structures. (B and C) Negative contrastation and diameter measurement of EVs secreted by *A. castellanii* isolated from (B) PYG (PYG-EVs, average diameter of 117.1 ± 73.3 nm as indicated by the axis thick and gray dashed line) and (C) glucose (glucose-EVs, average diameter of 117.7 ± 55.8 nm indicated by axis thick and gray dashed line) medium. On images (B and C), solid black arrows indicate the presence of nano-EVs (<50 nm); open arrows the presence of EVs ranging from 50–200 nm and white arrows, a population of large EVs (>200 nm). Displayed results are the average of 2 independent experiments.

Scanning electron microscopy of *A. castellanii* grown in PYG medium revealed the presence of membrane bound vesicles of circular shape and distinct sizes, composing a polydisperse population, as a result of evagination from the extracellular membrane (Figure 1A). The dimensions of these structures closely approximate dimensions of EV reported for many microorganisms.

### A. castellanii produces distinct vesicle populations depending on growth conditions

*A. castellanii* was grown in two distinct conditions, under ATCC recommended culture medium (712 PYG) and in media without any protein source (glucose medium), to recapitulate the nutritional stress undergone by *A. castellanii* in the aqueous and vitreous humor *in vivo*. After negative staining of EVs from both culture conditions, they were evaluated by transmission electron microscopy, and the dimensions of EVs measured. *A. castellanii* EVs from PYG medium (PYG-EVs) displayed a diameter ranging from 31.9 to 467 nm (picture inset, Figure 1B), with an average diameter of 117.1 ± 73.3 nm (Figure 1B; axis thick and gray dashed line). EVs obtained from *A. castellanii* grown in glucose medium (glucose-EVs) ranged from 33.7 to 303.2 nm (picture inset, Figure 1C), with an average diameter of 117.7 ± 55.8 nm (Figure 1C, axis thick and gray dashed line). Despite similar diameter averages at both growth conditions (*p* > 0.05), EVs from *A. castellanii* grown in PYG medium consisted in a more diverse population.

### Dynamic light scattering displays polydispersity in extracellular vesicle populations depending on medium composition

**Figure 2.**
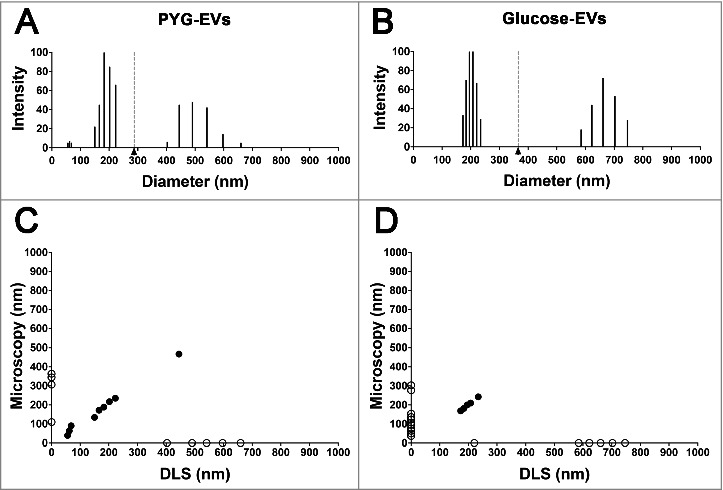
Characterization of the EVs’ populations secreted by *A. castellanii* by dynamic light scattering (DLS). The graphs are representative of the two replicates displaying similar results. EVs secreted and isolated from *A. castellanii* grown in (A) PYG (PYG-EVs, total average of 287.7 ± 154.8 nm, black arrow and gray dashed line) and (B) glucose medium (glucose-EVs, total average of 365.1 ± 224.6 nm, black arrow and gray dashed line). (C and D) Correlation of the diameters obtained by electron microscopy and DLS of EVs isolated from *A. castellanii* grown in (C) PYG (PYG-EVs) and (D) glucose medium (glucose-EVs). Open circles (○) indicates EVs populations identified in only one of the size measurement methodology, whereas closed circles (•) indicates EVs populations identified in both methods.

To confirm the results discussed above, we measured the size of larger number of EVs using dynamic light scattering (DLS) and their diameters were compared. PYG-EVs in solution displayed three major populations, 56.1 to 68.4 nm, 150.4 to 223.0 nm and 402.9 to 659.4 nm (total average of 287.7 ± 154.8 nm, Figure 2A). Glucose-EVs instead, displayed only two populations, with diameters ranging from 173.2 to 234.8 nm and from 585.1 to 746.5 nm (total average of 365.1 ± 224.6 nm, Figure 2B), slightly larger than EVs obtained from growth in PYG (p>0.05).

EVs diameters by microscopy and DLS were compared and correlated. PYG-EVs measurements by both methodologies significantly correlated (r = −0.53 and p = 0.022, Figure 2C), as common measurements were observed for the three populations identified. As for PYG, microscopy and DLS measurements of glucose-EVs correlated significantly (r = −0.44 and p = 0.040, Figure 2D), despite the lower number of equivalences among the total populations when compared to PYG-EVs, with common specimens detected for size populations around 200 nm.

### Lipid composition of A. castellanii extracellular vesicles

**Figure 3.**
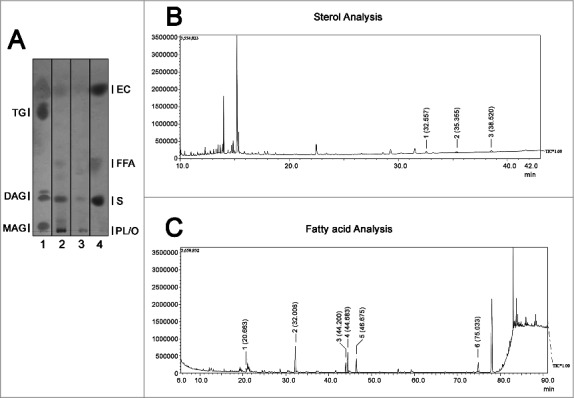
Evaluation of neutral lipid composition of EVs. (A) High performance thin liquid chromatography for neutral lipids of the EVs from *Acanthamoeba castellanii*. Lane 1 – Lipid standards (TG- Triglycerides −; DAG- Diacylglycerol; MAG- Monoacylglycerol), Lane 2- EVs secreted in PYG, Lane 3- EVs secreted in glucose medium and Lane 4- Lipid standards (EC- Esterified cholesterol; FFA- Free fatty acids; S- cholesterol; PL/O- Phospholipids. (B) GC-MS for determination of sterol composition in EVs of *A. castellanii*. Two biological replicates were analyzed with similar results. Peaks of interest are represented by numerals, followed by their retention time in the chromatogram. Peak 1 – [(3-β)-cholest-5-en-3-yl]oxy]trimethyl]-silane (rt = 32.557 min), Peak 2 – [(ergosta-5,7,22-trien-3β-yloxi)trimethyl]-silane (rt = 35.355 min) and Peak 3 -stigmasta-5,7,22-trien-3α-ol (rt = 35.520 min). (C) GC-MS for fatty acids present in the EVs of *A. castellanii*. Peaks of interest are represented by numerals, followed by their retention time in the chromatograph. Peak 1- methyl miristate (rt = 20.663 min), Peak 2- methyl palmitate (rt = 32.008 min), Peak 3- methyl linoleate (rt = 44.200 min), Peak 4- methyl oleate (rt = 44.683 min), Peak 5- methyl stearate (rt = 46.675 min) of and Peak 6- methyl erucate (rt = 75.033 min).

Total lipids extracted from *A. castellanii* EVs were qualitatively evaluated by high performance thin layer chromatography (HPTLC), (Figure 3A). Results shown are representative of 2 biological replicates (performed in duplicate). PYG-EVs of *A. castellanii*, displayed the presence of phospholipids, sterol, free fatty acid and steryl esters. Glucose-EVs displayed similar constitution, except for the absence of free fatty acids.

Since the lipid evaluation of PYG-EVs showed a more complex composition, we submitted this sample to GC-MS for the characterization of the sterol and free fatty acids species. Regarding sterol analysis, three major peaks were identified according to their retention time and MS fragmentations (Figure 3B and Supplemental figure 1A-C). Peak 1 was representative of a [(3-β)-cholest-5-en-3-yl]oxy]trimethyl]-silane (retention time(rt) = 32.557 min), whereas peak 2 was characteristic of a [(ergosta-5,7,22-trien-3β-yloxi)trimethyl]-silane (rt = 35.355 min) and peak 3, a stigmasta-5,7,22-trien-3α-ol (rt = 38.520 min). Regarding free fatty acids analysis, six major peaks were identified (Figure 3C and Supplemental figure 1D-I). Peak 1 (rt = 20.663 min) was characteristic of methyl myristate, peak 2 (rt = 32.008 min) of methyl palmitate, peak 3 (rt = 44.200 min) of methyl linoleate, peak 4 (rt = 44.683 min) of methyl oleate, peak 5 (rt = 46.675 min) of methyl stearate and peak 6 (rt = 75.033 min) of methyl erucate.

### Mass spectrometry data analysis and protein identification

EVs and EVs-free supernatant from either PYG or glucose were evaluated by mass spectrometry. The proteins identified in each of three biological replicate (samples 1–3) were analyzed using a Venn diagram to find the number of common proteins in each condition/ fraction. In PYG-EVs, 110 proteins were common in the three replicates (Supplementary Figure 2A), whereas 90 proteins were common in the three replicates of PYG-EVs-free supernatant (Supplementary Figure 2B). For glucose-EVs, 148 common proteins were found in the three replicates (Supplementary Figure 2C), and 71 proteins found for glucose-EVs-free supernatant (Supplementary Figure 2D). The Supplementary Figure 2E displays in each fraction, the number of total proteins identified summing the three replicates (100% in all cases), the number of common proteins identified in at least 2 samples (light colors) and common to the three biological replicates (dark colors). These common proteins in the triplicates of each individual fraction/ condition were used for cross comparison of the secretome fractions (EVs and EVs-free supernatants) of *A. castellanii* under rich (PYG) or nutritional stress (glucose medium). Considering the total secretome (EVs and EVs-free supernatants), a comparison between the two nutritional conditions was also performed.

### Secretome of A. castellanii under rich nutritional conditions

**Figure 4.**
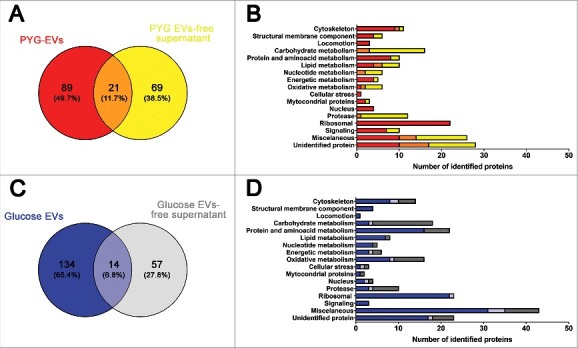
Identification and grouping of proteins in the secretome of *A. castellanii* grown in PYG (A and B) and under stress condition (glucose medium, C and D). (A) Venn diagram of the PYG secretome, comparing PYG-EVs (110 proteins) and PYG-EVs-free supernatant (90 proteins, from Supplementary Figure 2 A and B, respectively). From the 179 unique proteins identified, PYG-EVs exclusive proteins (89; 49,7%) are shown in red; proteins identified only in the EVs-free supernatant (69; 38.5%) are shown in yellow, and common proteins of both fractions are shown in orange (21; 11.7%) (B) Number of proteins identified exclusively in PYG-EVs are shown as red bars, whereas the number of proteins only in the EVs-free supernatant are shown in yellow and the number of proteins found in both fractions are shown in orange. (C) Venn diagram of glucose medium secretome, comparing glucose-EVs (148 proteins) and PYG-EVs-free supernatant (71 proteins, from Supplementary Figure 2 C and D, respectively), totalizing 205 unique proteins identified under nutritional stress (glucose medium). The number of proteins identified exclusively in glucose-EVs are shown in blue (134; 65.4%); proteins identified only in the glucose-EVs-free supernatant are shown in gray (57; 27.8%), and common proteins of both fractions are shown in light blue (14; 6.8%). (D) Number of proteins identified from each specific class exclusively in glucose-EVs are shown as blue bars, whereas number of proteins identified only in the glucose-EVs-free supernatant are shown in gray and number of common proteins are shown in light blue. Proteins identified in EVs, EVs-free supernatant or commonly present in both fractions from either PYG or glucose medium, and the class they belonged, are discriminated in Supplementary Tables 1 and 2, respectively.

PYG-EVs (110 proteins, as shown in Supplementary Figure 2A) and PYG-EVs-free supernatant proteins (90 proteins, as shown in Supplementary Figure 2B) identified proteins were grouped according to their functional class (www.uniprot.org) and compared. In PYG medium, a total of 179 unique proteins were identified by MS, with 89 proteins (49.7%) exclusively present in the PYG-EVs fraction, 69 proteins (38.5%) exclusively present in the PYG-EVs-free supernatant, and 21 proteins (11.7%) identified in both fractions (Figure 4A and Supplementary Table 1). The majority of proteins in both PYG-EVs and PYG-EVs-free supernatant belonged to the miscellaneous (14 vs. 16, respectively) and unidentified protein classes (17 vs. 18; Figure 4B). Comparing both fractions, proteins related to locomotion (4), cellular stress (2), nucleus (4) and ribosomal proteins (34) were found exclusively in the PYG-EVs. Furthermore, proteins related to cytoskeleton (EVs 10 vs. 2 EVs-free supernatant), structural membrane components (4 vs. 2), protein and amino acid metabolism (8 vs. 2), energetic metabolism (4 vs. 1) and signaling (7 vs. 3) were enriched in the PYG-EVs. Remarkably, the vast majority of proteases were found in the PYG-EVs-free supernatant (12), from which one was also detected in PYG-EVs. Also, proteins related to carbohydrate (3 vs. 13), nucleotide metabolism (2 vs. 6) and oxidative metabolism (2 vs. 5) were mostly present in the PYG-EVs-free supernatant.

### Proteins secreted by A. castellanii under nutritional stress

Glucose-EVs (148 proteins as shown in the Supplementary Figure 2 C) and Glucose-EVs-free supernatant (71 proteins, as shown in the Supplementary Figure 2 C) fractions of the secretome of *A. castellanii* were also compared. From the total of 205 unique proteins, 134 (65.4%) were present exclusively in glucose-EVs, 57 proteins (27.8%) were detected only in glucose-EVs-free supernatant and 14 proteins (6.8%) were shared by both fractions (Figure 4C and Supplementary Table 2). As observed with the proteome under rich nutritional conditions, the majority of EVs and EVs-free supernatant secreted proteins by *A. castellanii* under stress belonged to miscellaneous (35 vs. 12, respectively) and unidentified protein classes (18 vs. 6; Figure 4D). Comparing both fractions, proteins related to structural membrane components (4), locomotion (4) and signaling (3) were found exclusively in the glucose-EVs fraction. Proteins from cytoskeleton (10 vs. 6), protein and amino acid metabolism (16 vs. 6), lipid metabolism (7 vs. 1), nucleotide metabolism (4 vs. 1), nucleus (3 vs. 2) were predominantly present in glucose-EVs fractions after *A. castellanii* growth under nutritional stress. As in PYG medium, proteins related to the metabolism of carbohydrate (4 vs. 15) and proteases (4 vs. 7) were mostly enriched in the glucose-EVs-free supernatant. Regarding ribosomal proteins, the vast majority was found in EVs (22), and one common protein within the two fractions.

### Comparison of A. castellanii secretome in PYG and nutritional stress conditions

The total secretome (EVs + EVs-free supernatant) under each specific growth conditions was compared (PYG 179 proteins and glucose 205 proteins), with 127 proteins (38.3%) exclusively expressed in the PYG secretome, 153 proteins (46.1%) exclusively expressed in glucose secretome and a total of 52 proteins (15.7%) were commonly identified (Supplementary Figure 7A). In the *A. castellanii* secretome under stress conditions, there were a higher number of proteins involved in protein and amino acid (PYG 10 vs. 22 glucose) and oxidative metabolism (6 vs. 16, respectively), as well as proteins related to cellular stress (1 vs. 3; Supplementary Figure 7B). However, proteins involved in locomotion (3 vs. 1) and signaling (10 vs. 3) were mostly present when *A. castellanii* was grown under abundance of protein sources.

### Secretome and carbohydrate metabolism regulation in A. castellanii

Despite the similarity in the carbohydrate sources in both *A. castellanii* growth conditions and the overall undistinguishable number of proteins related to carbohydrate metabolism (PYG 16 vs. 18 in glucose alone, with 3 proteins commonly detected; Supplemental Figure 3A), we explored the qualitative differences on carbohydrate usage in both conditions, considering the total secretome (EVs and EVs-free supernatant). A metabolic map displaying the carbohydrate metabolism enzymes detected was constructed (Supplementary Figure 3C).

In both conditions, glycosyl hydrolase was the most prevalent class, with one common entity, as they were involved in the utilization of complex sugar sources. Besides, an α-amylase and the glycolytic pathway enzyme enolase were commonly expressed under the two growth conditions. Exclusively in the secretome of PYG, the enzymes β-glucosidase, β-galactosidase, xylosidase, α- and β-mannosidase were identified and involved in the breakdown of complex sugars. Specifically in glucose medium, proteins such α-1,4-glucan phosphorylase and glucan (α-1,4) branching enzyme were identified. Yet in glucose medium, enzymes from glycolysis, such as glucose-6-phosphate isomerase and triose phosphate isomerase were identified. Enzymes involved in the metabolism of phosphoenol pyruvate (PEP), PEP carboxylase and PEP carboxykinase were also identified, along with two malate dehydrogenase enzymes from the Krebs. In this condition, a transaldolase enzyme involved in the pentose-phosphate pathway, was also observed.

### Secretome of A. castellanii and protease identification

The proteases indentified in the EVs and EVs-free supernatant of both growth conditions were grouped into five mains classes as shown in Supplementary table 3. No statistical differences were observed between both growth conditions (p>0.05). However, considering all instances, serine proteases was the most abundant class (9), followed by metalloproteases (6), aspartic proteases (2) and cystein proteases (1). To further confirm the entities from these classes, EVs and EVs-free supernatant were analyzed by gel electrophoresis and their protease activity determined, as the classes of proteases function better at specific ranges of pHs (serine proteases ≥7.0, metallo ≥7.0, 3.0≤ aspartic ≤ 5.0, cysteine ≥7.0) [58]. For all the fractions characterized, protease activity was observed at all pHs tested; however the vast majority of proteins and highest activity was observed at pH 9.0, due to optimum conditions for serine, metallo and cysteine protease activity, being the first two the most abundant protease classes found (Supplemental Figure 4).

### EVs are readily taken up by mammalian cells

**Figure 5.**
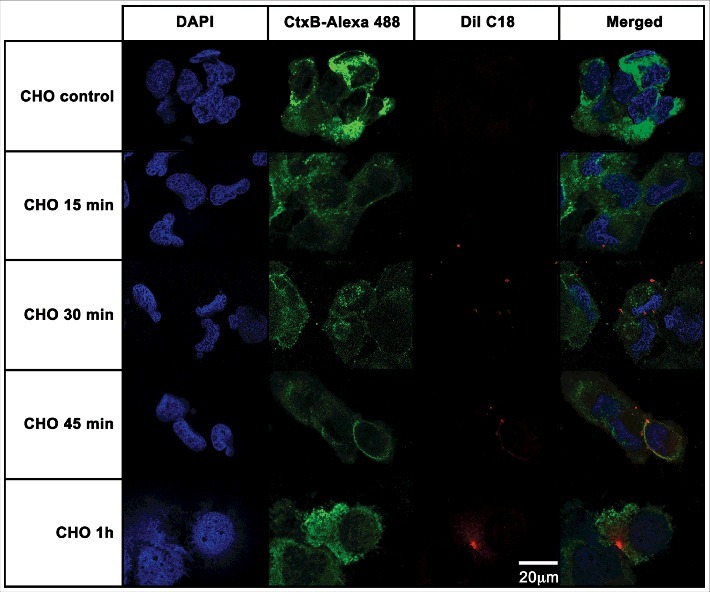
CHO epithelial cells internalization of EVs from *A. castellanii*. EVs were stained with DiI C18 (red-stained) and incubated with the CHO for different time points. CHO nuclei were stained with DAPI (blue) and the CtxB- Alexa 488 (green) was used to stain the GM1 ganglioside, a lipid raft marker located on the cell membrane. *A. castellanii* EVs (DiIC18 red labeled) co-localized with the lipid rafts, suggesting association of GM1 on the CHO internalization of EVs. At early time points, EVs can be found in association to the plasma membrane of CHO cells; at 1 h, EVs seem to be distributed or disseminated through the cytoplasm of CHO cells. Results are representative of at least 10 different fields.

To verify whether the EVs from *A. castellanii* could be recognized by the host, these membranous compartments were stained with DilC18 (1,1'-Dioctadecyl-3,3,3', 3'-Tetramethylindocarbocyanine Perchlorate) and given to the mammalian cells. This lipophilic dye intercalates into the membrane phospholipid bilayer, and has been used to label EVs in several instances [31,59,60]. Binding and internalization of EVs of *A. castellanii* by CHO (epithelial lineage) or T98G (glial lineage) cells were evaluated by confocal microscopy followed by deconvolution analysis. For CHO cells, after 15 min of contact, EVs could be found in association to the cell membrane (Figure 5, second row), as the red-fluorescent DiI C18 labeled EVs co-localized to the green fluorescent CtxB, which labels GM1, indicating the involvement of host cells lipid raft in the EVs cell internalization (Figure 5, second row, merged images). After 30 min of exposure, CHO cells displayed more cell membrane-associated EVs (Figure 5, third row). Following 45 min, EVs could still be found in association to CHO cell membranes or randomly distributed in the cytoplasm (Figure 5, forth row). After 1 h, vesicles were completely internalized and disseminated through the cytoplasm (Figure 5, last row).

**Figure 6.**
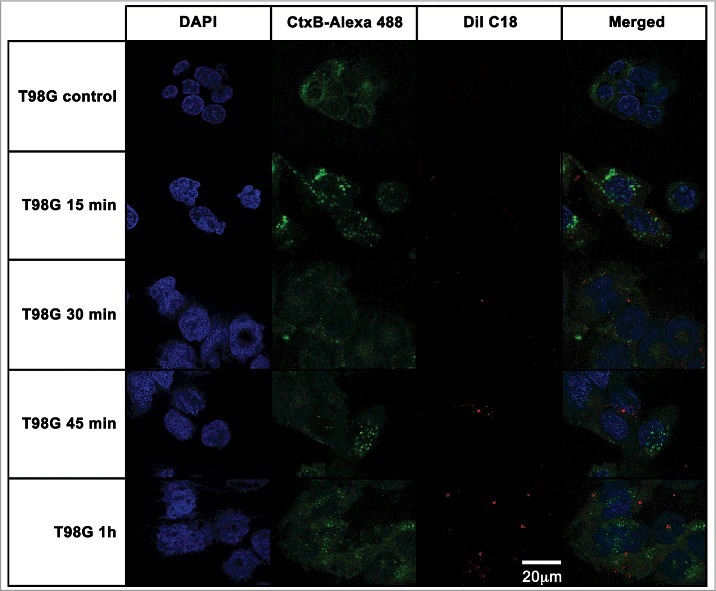
T98G glioblastoma cells internalization of EVs from *A. castellanii*. EVs were stained with DiI C18 (red-stained) and incubated with the CHO for different time points. T98G nuclei were stained with DAPI (blue) and the CtxB- Alexa 488 (green) was used to stain the GM1 ganglioside, a lipid raft marker located on the cell membrane. *A. castellanii* EVs co-localized with the lipid rafts, suggesting association of GM1 on the T98G internalization of EVs. At early time points, EVs are associated to the membrane of T98G cells; at 1 h, EVs seems to be randomly distributed through the cytoplasm of T98G cells. Results are representative of at least 10 different fields.

For T98G cells (Figure 6), the kinetics of *A. catellanii* EVs association followed a similar pattern as observed for CHO cells, with T98 cell membrane-associated EVs at 15 min and 30 min (Figure 6, second and third rows, respectively) and EVs distributed through the cytoplasm at 45 min (Figure 6, forth row). However, after 1 h, vesicles were still compartmentalized within the cytoplasm of T98G cells (Figure 6, last row).

### A. castellanii secretome toxicity to mammalian cells is dependent on protease activity

**Figure 7.**
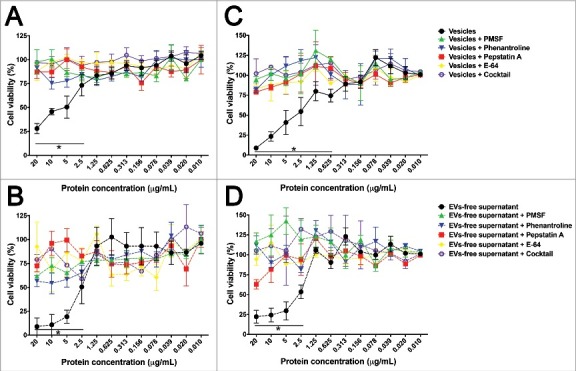
Effects of *A. castellanii* secretome on viability of mammalian cells. Cells were incubated with decreasing concentrations of *A. castellanii* secretome (from 20 to 0.010 µg/mL, expressed in total protein content). Cell viability was calculated by the ratio of absorbances of (treated cells)/(untreated negative control). (A-B) Cell viability of CHO cells determined by the MTT assay upon 6 h incubation with (A) EVs and (B) EVs-free supernatant (symbols and solid lines). (C-D) Cell viability of T98G cells determined upon 6 h incubation with (A) EVs and (B) EVs-free supernatant (symbols and dashed lines). PMSF- serine protease inhibitor, phenantroline – metalloprotease inhibitor, pepstatin A- aspartic protease inhibitor, E-64- cystein protease inhibitor and cocktail – complete mini-tabs (Sigma-Aldrich). *p < 0.05.

Due to ability of CHO and T98G cells to uptake EVs and the presence of several potential virulence factors in the secretome of *A. castellanii*, their effects on mammalian cells were tested (Figure 7). Controls with BSA, PYG, irrelevant or disrupted *A. castellanii* EVs had no effect on either CHO or T98G cells viability. For CHO cells, a dose-dependent effect of EVs was observed, with cell viability of 28% when 20 µg/mL was used, and cytotoxic effect observed with concentrations as low as 2.5 µg/mL (73% viability, Figure 7A). EVs-free supernatant also demonstrated a dose-dependent effect, with 8.9% viability at 20 µg/mL and cytotoxic effects with concentrations as low as 2.5 µg/mL (50% viability; Figure 7B). In both cases, inhibition of proteases with subclass specific protease inhibitors protected the cells against the damaging activity of both EVs and EVs-free supernatant.

For glioblastoma T98G cells, EVs also induced a dose-dependent effect on cell viability, with viability dropping to 8.8% at concentrations of 20 µg/mL and cytotoxicity observed with concentrations higher than 0.625 µg/mL (74% viability, Figure 7C). EVs-free supernatants caused a viability reduction to 22% at concentrations of 20 µg/mL, with a little increase to 53% viability when concentrations of 2.5 µg/mL were used (Figure 7D). For these T98 cells, inhibition of proteases also protected the cells against damage.

### Apoptosis/necrosis of mammalian cells after exposure to the secretome of A. castellanii

**Figure 8.**
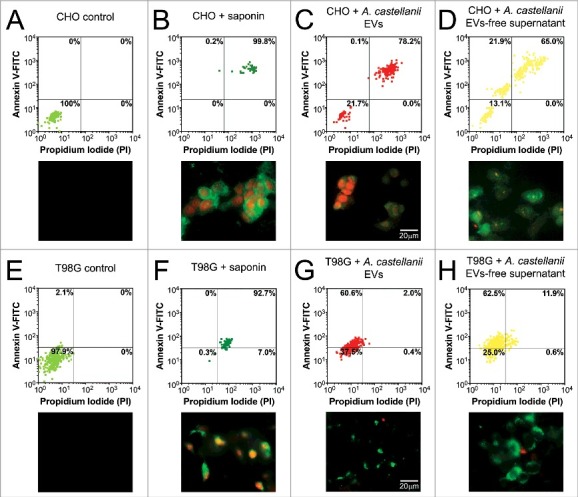
Evaluation of cell death upon treatment of CHO and T98G cells with EVs or EVs-free supernatant. Cells were stained with the Annexin V-FITC (green)/ PI (orange) kit, fluorescence images were recorded and the intensity of cell analyzed for each channel. (A-D) CHO evaluation upon treatment with *A. castellanii* EVs. (A) untreated CHO control, (B) saponin treated CHO, (C) EVs treated and (D) EVs-free supernatant treated CHO cells. (E-H) T98G treatment with *A. castellanii* EVs. (E) untreated T98G cells, (F) saponin treated T98G, (G) EVs treated and (H) EVs-free supernatant treated CHO cells. Pictures bellow each graph are representative of the microscopy images. Saponin treated CHO or T98G cells, and CHO treated with EVs or EVs-free supernatant displayed double positivity (PI^+^/Annexin V^+^), with strong stain of nuclei (orange) and phosphatidylserine on cell membrane (green), suggesting a necrotic process. T98G cells treated with EVs or EVs-free supernatant displayed a single stain (PI^−^/Annexin V^+^), suggesting apoptosis.

To assess whether the isolated fractions induced cellular necrosis or apoptotic death, CHO and T98G cells were double-stained with PI to probe loss of cell membrane integrity of necrotic cells and annexin V, which stains phosphatidylserine residues on the surface of early apoptotic cells. Both untreated controls of CHO (Figure 8A) and T98G (Figure 8E) cells displayed 100% and 97.9% of viable unlabeled cells (PI^−^/ annexin V^−^, respectively), whereas saponin treated CHO and T98G cells were 99.8% and 92.7% double-stained (PI^+/^ annexin V^+^), indicating necrosis (Figures 8B and F, respectively). EVs-treated CHO cells displayed 72.8% of necrotic cells (PI^+^/ annexin V^+^, Figure 8C). In turn, treatment with EVs-free supernatant resulted in a mixed population of 65% necrotic (PI^+^/ annexin V^+^) and 21.9% apoptotic cells (PI^−^/ annexin V^+^, Figure 8D).

For T98 cells, EVs treatment resulted in 60.6% apoptotic cells (Figure 8G). As observed for CHO cells, T98G cells treatment with EVs-free supernatant displayed a mixed population of 11.9% necrotic and 62.5% apoptotic cells (Figure 8H).

## Discussion

The first shedding of EVs in protozoa was described in trypomastigotes derived from *T. cruzi* culture [61]. Subsequently, EVs secretion was described for other trypanosomatid species such as *T. brucei* [44,45] and *Leishmania* sp [42,43]., *Plasmodium* spp [41]., nematodes [48-50], trematodes and the amoeba species *D. discoideum* [52]. Hence, production of EV appears to be a common mechanism for secretion among the protozoa.

The EVs described for these protozoa are in most reports, exosomes with a diameter ranging from 30 to 100 nm, with a wide variety of functions, from playing a role in the host-parasite relationship [45,50] to inducing carcinogenic process, due to the presence of micro RNA and delivery through the EVs to the host cell environment [62]. In *D. discoideum*, Taticheff and collaborators [52] described the presence of three EVs populations: smaller than 50 nm nanovesicles, capable of carrying molecules from the Golgi to outside of the amoeba, between 50–150 nm and vesicles larger than 150 nm. Trophozoites of *A. castellanii* release bilayered spherical structures with a larger diameter and greater polydispersion than those described for other parasites, specifically when compared to the EVs described in *T. cruzi*, which presents vesicles ranging from 20 to 80 nm in diameter [61] or *D. discoideum*, with vesicles smaller than 200 nm in diameter [52]. However, as we have used differential centrifugation for the preparation of large amounts of EVs, this could result in the recovery of different EVs populations compared to other techniques [52,61,63], such as size exclusion or filtration. Additionally, its main limitation is the co-precipitation of these membranous structures with large protein aggregates, nucleosomal fragments or apoptotic bodies, which could result in lower sample purity [64,65].

EVs heterogeneity was confirmed by electron microscopy imaging and DLS; however, the DLS has detected EVs > 400 nm, which could be the result of the association of two or more EVs, detected as a single particle by this laser-beam based technique [66,67]. Such large structure may form during collection of EVs by centrifugation and pelleting. However, both methodologies displayed correlation, making our data in relation to EVs dimension even more accurate.

Little is known about the mechanism by EVs are secreted from fungi, bacteria and protozoa. In fungi, for example, the presence of molecules such as lipids, glycolytic pathway enzymes, proteases and nuclear material have already been described in several yeasts [37–45]. More specifically for the genus *Acanthamoeba*, there is no description of the molecular composition of EVs. However, in the present work, we describe the secretion of molecules that were also described in EVs from distinct microbial origin. GC-MS was performed for the analysis of EVs' sterols, which identified sterols such as the cholesterol derivative [(3-β)-cholest-5-en-3-yl]oxy]trimethyl]-silane, an ergosterol analog [(ergosta-5,7,22-trien-3β-yloxi)trimethyl]-silane and stigmasta (stigmasta-5,7,22-trien-3α-ol) and fatty acids with carbon chains ranging from 14 to 22 carbons. Ergosterol is a typical sterol isolated from fungal cell membranes, whereas stigmasta is commonly found in plant roots and algae [68]; both sterol have been described in total extracts of *A. polyphaga* [69]. The presence of stigmasta appears to be linked to metabolism control. For instance sterols from *Anacardium occidentale*, were intravenously administered in dogs for glycemic stabilization [68]. Another possible function of long chain fatty acids described in *A. castellanii* could be related to some step in membrane remodeling, as described of *A. polyphaga* [70].

The dearth of purification techniques for the isolation and standardized protocols for the characterization of EVs has impaired the best understanding and elucidation of the functional properties and comparison of EVs from distinct organisms. However, to validate our results, we compared the proteins obtained in EVs of *A. castellanii* to previously published EVs vesicles characterization of *T. cruzi* [71], *L. major* [43] and *Plasmodium* sp [72]. Some similar markers were found, such actin (L8HCZ7 as found in *T. cruzi, L. major* and *Plasmodium* sp.), heat shock proteins hsp83 (L8H6T6, as found in *T. cruzi* and *L. donovani*) and hsp60 (L8GUV2 as found in *T. cruzi)*, histone 2B (L8H4F8 as found in *L. major* and *Plasmodium* sp.) and serine proteases (L8H1H6 and L8HEC6 as found in *T. cruzi*). However, a more elaborated comparison of protozoan EVs proteins needs to be performed.

When comparing the secretome of the distinct growth conditions, we could clearly observe a compartmentalization of proteins in each of the distinct fractions. This compartmentalization alone raises the hypothesis of a directed participation of the EVs in cell-cell communication, due to the presence of cell signaling molecules, as also hypothesized for *D. discoideum* [73–75]. Such hypothesis is supported by our data, which has shown that the number of proteins with biological functions related to cell signaling, ribosomal proteins and locomotion are exclusive to EVs. In turn, the EVs-free secretome, carrying carbohydrate metabolism related and depolymerases proteins, would be directed to the extracellular digestion and nutrient acquisition.

*A. castellanii* secreted proteins in PYG rich medium were related primarily to locomotion and signaling pathways. Specifically, *A. castellanii* adaptation to adverse conditions under nutritional stress (glucose alone) resulted in the secretion of molecules related to protein and amino acid metabolism, cellular stress and oxidative metabolism. The nutritional stress also became determinant in the metabolism of carbohydrates used by the amoeba, making it even more evident that the PYG medium for later trials mimics an ideal culture condition for amoebae. Besides the detection of glycosyl hydrolases and amylases in both culture conditions, the profile of detected enzymes revealed an enriched set of carbohydrate hydrolyzing enzymes in conditions of protein starvation, including a α-1,4-glucan phosphorylase and glucan (α-1,4) branching enzyme, for starch and glycolipids/glycoproteins digestion respectively, in order to increase energy acquisition/production.

The glycolysis enzyme enolase was also identified in EVs secreted by *A. castellanii* trophozoites in both culture media. Enolase is usually induced during cyst formation, but its role in the cyst cellulose inner layer polymerization is unclear; EVs could be strictly involved in the trafficking of this enzyme to the cell surface [76]. We did not observe cysts after 48 hours of growth in the glucose media (data not show); however these culture conditions are unstable for the growth of the trophozoite phase for longer periods. Remarkably, when *A. castellanii* is cultivated in glucose medium, they secreted enzymes related to pentose phosphate shunt for the glucose *de novo* synthesis, despite availability of this carbon source. All this plasticity in the metabolism of carbohydrates empowers amoeba to use different resources to be able to succeed during the process of colonization of distinct niches.

The importance and role of secreted proteases in the pathogenesis of infectious diseases has been established for several microorganisms [27–30,35–41,45], including different species of parasites, such as *Trichomonas vaginalis* [77], *Giardia lamblia* [78], *P. falciparum* [79] and *Leishmania* sp [42,43].. For instance, *Entamoeba histolytica* secretes cysteine proteases (EhCP112) that degrades the protective mucus barrier of the intestine and breaks epithelial cells monolayers. Simultaneously, it promotes intestinal invasion and extra-intestinal infection [80,81]. *A. castellanii* produces serine, cysteine and metalloproteases, and extracellular protease activities are increased in pathogenic *Acanthamoeba* strains [13,82]. T4 genotype of *A. castellanii* secretes mainly serine proteases, whereas other nonpathogenic amoebae are able to produce metallo and cysteine proteases [83]. This class of proteins plays a role in both pathogenicity [24,25] and amoeba differentiation between cist/trophozoite [76,83]. Proteases were remarkably abundant in the secretome of *A. castellanii*.

Our data also demonstrates that both fractions of the secretome of *A. castellanii* are able to reduce cell viability of epithelial cells (CHO) and blood-brain barrier cells (T98G). When CHO cells are incubated with EVs, they initially accumulate on the surface and their contents diffuses to the cytoplasm causing cell death by necrosis (PI^+/^ annexin^+^); EVs-free supernatant, in the other hand, induced a mixed population of major necrotic and some apoptotic cells. T98G gliobalstoma cells treatment with either EVs, also resulted in vesicle accumulation on the surface, with subsequent phagocytosis; however random accumulation of these structures can be observed in the cytoplasm without content diffusion and subsequent cell death occurs by apoptosis; EVs-free supernatant, in turn, caused death mainly by apoptosis, but a small population of necrotic cells could be also observed. As EVs displayed cytotoxicity to either CHO or T98 mammalian cell models tested, cell death mechanism was lineage dependent (necrotic and apoptotic, respectively). But commonly to both lineages, inhibition of proteases resulted in no damage to these cells, as we concluded that these enzymes constitute one of the main virulence factor directly implicated to host tissue damage.

The presence of proteases, kinases and glycosidases in EVs could contribute with the infection process in humans, since these molecules enable the parasite to establish and colonize host tissues. EVs may themselves be involved in the modulation of the interaction between *A. castellanii* itself and host cells, and mediate the tropism of certain strains of *A. castellanii* to retinal tissue (ocular keratitis) and central nervous system [5,6,11,14,20–23]. Huang and collaborators showed that proteins secreted by *A. castellanii*, induced the detachment of mammalian cells and assigned this effect to the M20/M25/M40 aminopeptidase superfamily proteins. These detached cells floated and were then ingested and killed by amoeba cells [84]. These proteins were not found in the EVs or EVs-free supernatant, but other molecules present in the secretome of *A. castellanii* could induce similar functions. The secretome of *A. castellanii* may also participate in immunomodulation, being able to induce response patterns cytokines and recruitment of cells, suggesting also that there may be a difference between the secretomes of the different genotypes.

The hypothesis that *A. castellanii* serves as a host that selects for virulence trait by preying upon parasitizing microorganism is attractive, but still remains to be elucidated [85]. Recently, the interaction of *A. castellanii* and *C. neoformans* was demonstrated to have an impact on virulence, as a more virulent phenotype of *C. neoformans* expressing distinct available surface polysaccharides, such as chitin and distinct virulence traits, could be isolated upon interaction with *A. castellanii* [*86*]. We hypothesize that one mechanism for imposing selection pressures on fungi might be through the digestion of cell wall surface sugars, since glucosidases, xylosidases and mannosidases are present in the *A. castellanii* secretome, facilitating the fungal phagocytosis by the amoeba and modulating positively their adaptation to its intracellular milieu, with subsequent production of amoeba resistant strains, potentially more pathogenic.

In summary, we provide the first description and characterization of EVs from *A. castellanii*. Our results shed new light for the development of new pharmacological options that interfered with EVs secretion, as reducing the damage provoked by *A. castellanii* to tissues, they could be useful therapeutic agents for amebiasis.

## Materials and methods

### Culture of Acanthamoeba castellanii

*A. castellanii* (ATTC 30234) were obtained from the American Type Culture Collection (ATCC, Manassas, VA). Trophozoites were cultured as adherent cells under axenic condition until reached confluence in peptone-yeast extract-glucose (ATCC 712 medium PYG; 20.0 g/L proteose peptone, 1.0 g/L yeast extract, supplemented with 0.4 mM CaCl_2_, 4 mM MgSO_4_, 2.5 mM Na_2_HPO_4_, 2.5 mM KH_2_PO_4_, 3.4 mM sodium citrate, 0.05 mM Fe(NH_4_)_2_(SO_4_)_2_, pH 6.5, 100 mM glucose, filter-sterilized). The tissue culture flasks were kept at 28°C in the absence of light. *A castellanii* was subcultured every 48h and, under these conditions, >99% of amoeba were trophozoites. For *A. castellanii* cultivation under nutritional stress, trophozoites were kept under the same conditions as described above, in a similar medium without addition of peptone and yeast extract, denominated glucose medium.

### Culture of animal cells

Chinese hamster ovary (CHO) cells were obtained from the Cell Bank of Rio de Janeiro and cultivated in HAM F-12 medium supplemented with 1.2 g/L NaHCO3, 5% fetal calf serum (Cultilab), 2% non-essential amino acids and 1% penicillin/streptomycin (LifeTechnologies). T98G cells were cultivated in high glucose DMEM medium supplemented with 5% FCS, 1% non-essential amino acids and 1% penicillin/streptomycin. Cells were maintained at 37^o^C and 5% CO_2_ and passaged every two days until use.

### Scanning electron microscopy

*A. castellanii* (1 × 10^6^ cells) was cultured in PYG on the surface of a 13 mm diameter glass cover slip for 3 h at RT. After, cells were washed with 1 M sodium cacodylate buffer, followed by a fixation step using 1 M sodium cacodylate buffer and 2.5% glutaraldehyde for 2 h at RT. Fixed cells were treated with a 1% osmium tetroxide for 30 min, then dehydrated in serially increasing acetone concentrations (30%, 50%, 70%, 90% and 100%). We performed the critical point drying followed by metallization with gold, where the sample received a thin layer of approximately 52 nm coating. Samples were then analyzed in a JEOL JMS-6490LV microscope [87].

### Secretome preparation

After 48 h of cultivation in both PYG and glucose medium, *A. castellanii* culture supernatants were collected and centrifuged at 1,100 × *g* for 10 min. The cell pellet was discarded and remaining trophozoites and cell debris were removed by an additional step of supernatant filtration using a 0.8 μm membrane filter (Merck Millipore). The filtered material was collected in sterile glass flasks and conditioned at 4–8°C until further processing for extracellular vesicles (EVs) isolation. One liter of cell-free supernatants was concentrated approximately 20-fold using a 10 KDa cut-off membrane in an Amicon ultrafiltration system (Merck Millipore) and then ultracentrifuged at 100,000 *× g* for 1 h at 4°C. Supernatants were collected and saved as soluble fractions (or EVs-free supernatant). Pellets containing EVs were further washed twice with a 0.22 μm filtered phosphate-buffered saline (PBS, 140 mM NaCl, 2.7 mM KCl, 1.5 mM KH_2_PO_4_, 17 mM Na_2_HPO_4_, pH 7.2)(Merck Millipore), and ultracentrifuged at 100,000 *× g* for 1 h at 4°C. The protein concentrations of EVs and EVs-free supernatant fractions from both growth conditions were determined by the bicinchoninic acid (BCA) method according to manufacturer´s instructions (Life Technologies).

### Dynamic light scattering (DLS)

EVs were diluted in filtered PBS and their size distribution was determined by Quasi-Elastic Light Scattering in a 90Plus/BI-MAS Multi Angle Particle Sizing analyzer (Brookhaven Instruments, Holtsville, NY), set for detection of populations ranging from 10–1500 nm. Multimodal size distribution analysis of particles was calculated from the values of intensity weighted sizes obtained from the non-negatively constrained least squared (NNLS) algorithm [37]. Measurements of two biological replicates were performed at 25°C, in duplicates.

### Transmission electron microscopy

EVs from *A. castellanii* were negatively stained to determine their dimensions as described elsewhere [88]. For this procedure, 10 µL of EVs suspension were deposited on copper grids (200 mesh) and air-dried. Grids were treated with Formvar 0.3% and charred. EVs were negatively contrasted with 1% (m/v) uranyl acetate. The micrographs were taken in an electronic microscope JEOL 1200EX of 80 kV and images analyzed by Image J (NIH, Bethesda) in order to determine the vesicles diameter. At least 200 vesicles were analyzed in the 3 grids prepared for each of 2 biological replicates from each growth condition.

### Lipids analysis

Lipid composition of EVs was resolved by high performance thin layer chromatography (HPTLC). Initially, 100 µg of EVs from two biological replicates were partitioned in chloroform: methanol: water (2:1) [57,89]. Then the lower phase was applied to a TLC silica gel plate (Merck). For neutral lipid analysis, the lipid extract was resolved in a hexane: ether: acetic acid (80:40:2 v/v/v) and plates developed with a solution containing 0.5 mg/mL of FeCl_3_ in 5% (v/v) acetic acid and 5% (v/v) sulfuric acid. Plates were heated at 100ºC for 3–5 min and lipid bands were compared to commercial standards of triglyceride, diacylglyceride, monoacylgrlyceride, free fatty acid, sterol and esterified cholesterol (Sigma-Aldrich).

Sterol and free fatty acids (FFA) were also characterized by chromatography associated with mass spectrometry (GC-MS) in the facility at the Federal University of Rio the Janeiro, using a total of 20 µg of lipids extract from two independent replicates of isolated EVs. Extraction of lipids was carried out in duplicate using a mixture containing chloroform: methanol: water (0,5:1:0,4 v/v/v). Suspensions were vortexed until the appearance of two or more phases; methanol was added until the solution became homogeneous. Samples were vortexed every 5 min for 1 h and then centrifuged at 1,100 ×*g* for 20 min. The supernatant was collected and reserved. The pellet formed was subject to the same procedure described above and the supernatant was pooled with the previous one. Finally, 1 mL of chloroform: water (1:1) was added, fractionating the material in 2 phases, which was then vortexed and centrifuged at 1,100 ×*g* for another 30 min. At the end of the extraction, the organic phase extracted from our samples was collected [90].

For the saponification and derivatization, the samples were dried in N_2_ [91], suspended in 1 mL of 25% alcoholic potassium (25 g KOH in 35 mL H_2_O, supplemented with ethanol for 100 mL) for 1 min and directly subjected to 85°C heating for 1 h in a water bath. Upon cooling the samples at RT, a mixture of 1 mL of distilled H_2_O and 2 mL of heptane was added. The sample was vortexed, centrifuged at 1,100 ×*g* for 20 min and dried in a chemical hood with N_2_. For signaling, 50 µL of BSTFA: TMCS (99: 1) and 50 µL of pyridine were added to each sample and heated at 65°C for 1h. Finally, 1 µL was injected into a Shimadzu GC-MS model GP2010 Plus GC-MS, coupled to an HP Ultra 2 (5% Phenyl-methylpolysiloxane), Agilent (25 mx 0.20 mm X 0.33 µm). The injector was maintained at 250°C, splitless mode. The temperature of the column was raised to 50–270°C with a heating rate of 18°C/ min, and 270–300°C with a heating rate of 1°C/ min and then maintained for 6 min. Helium was used as drag gas with linear velocity of 33.0 cm/sec. A detector containing an electron ionization source (EI-70 eV) and a quadrupole mass analyzer, operated in sweeps of 40 to 600 u.m.a. was used for the detection by mass spectrometry. The interface and the ion source were maintained at 280°C. Identification of the constituents of the mixture was made by comparing their mass spectra with those of the NIST05 library [92].

### Liquid chromatography- tandem mass spectrometry (LC-MS/MS)

Preparation of protein samples for mass spectrometry analysis was carried out according to previously described protocols [93]. For mass spectrometry, we produced three biological replicates of either EVs or EVs-free supernatant from each growth condition (PYG or glucose) and the analysis of each sample was performed in duplicates. Initially, 100 μg of each sample was dried in a speed vac (Eppendorf), dissolved in 18 µL of 7 M Urea/ 2 M thiourea, and added of 2 µL 1 M HEPES. Samples were reduced by adding 2.2 µL of 100 mM 1,4-dithiothreitol and incubated at 30ºC for 1h. After, 2.5 µL of 400 mM iodoacetamide was added and samples incubated for 30 min in the dark at RT. A volume of 175 µL of MS compatible water (Tedia Corporation) was added to dilute the urea /thiourea, and trypsin (Promega) diluted in acetic acid was added to a final concentration of 0.1 µg/μL. Samples were incubated overnight at 37ºC. Then, 2 µL of 10% TFA was added to a final concentration of 0.1%, and the samples cleaned up with an Ultra micro Spin Column (Harvard Apparatus). After drying the samples in speedvac, tryptic peptides were re-dissolved in 10 µL of 0.1% trifluoroacetic acid (TFA) and the mixture (4 µL) was loaded onto an in-house packed 15 cm x 75 μm column filled with 3 μm ReproSil C18 resin (Dr. Maisch GmbH) using the NanoLC-Ultra nanoliquid chromatography system (Eksigent Technologies). Peptides were first loaded onto a trap column (20 × 0.2 mm) packed with Reprosil-Pur 120 C18-AQ 5 µm particles (Dr. Maish, Germany). After 4 min washing with solvent A [H_2_O: acetonitrile (95:5, v/v), 0.1% (v/v) formic acid (FA)] peptides were eluted during the gradient on a 150 × 0.075 mm column packed with C18 Reprosil Gold 300 3 µm particles (Dr. Maish) at a flow rate of 300 nL.min^−1^. Gradient was 5–35% of solvent B [H_2_O: acetonitrile (5:95, v/v), 0.1% (v/v) FA] for 110 min, 35–95% B for 20 min and 90% B for 10 min; equilibration was carried out for 20 min at 5% B. For sample analysis in the LTQ-Orbitrap (ThermoFisher), the mass spectrometry DDA cycle consisted of a survey scan acquired in one μscan within the range of m/z 300–1800 at target mass resolution of 60,000 FWHM (full width at half-maximum) and target value of 1E06 ions. Survey scan was followed by MS/MS fragmentation of the ten most abundant multiply charged precursors. Spectra were acquired in one μscan under the normalized collision energy of 35% and target value of 1E04 ions in the linear ion trap (ion selection threshold 400 counts; precursor ions isolation width 4 a.m.u.). Activation parameter q = 0.25 and activation time of 30 ms were applied. Previously fragmented precursors were dynamically excluded for 90 s. Data were acquired using the Xcalibur software (version 2.0, Thermo) LC-MS/MS runs were saved as .raw files.

### Mass spectrometry data analysis

MS/MS spectra in .raw format were processed in PattrernLab for Proteomics software v.4.0 (PMID: 2658470-Computacional Proteomics- Fiocruz) Peptide sequence matching (PSM) was performed using the Comet algorithm (PMID: 23148064). The coding sequences present in the genome of *A. castellanii* were downloaded from Uniprot (www.uniprot.org) and used as database. The search was performed considering the following parameters: (1) two trypsin missed cleavages allowed, (2) precursor peptide mass tolerance of 10 ppm, (3) MS/MS fragment mass tolerance of 0.6 Da, (4) cysteine carbamidomethylation as fixed modification and (5) methionine oxidation as variable modification. For protein identification we considered using a false discovery rate (FDR) set to 1%.

### Identified proteins in PYG and glucose EVs and EVs-free supernatant

The list of identified proteins in the three biological replicates of each specific condition (PYG-EVs, PYG-EVs-free supernatant, glucose-EVs or glucose-EVs-free supernatant) were used to construct Venn diagrams (BioinfoGP, CNB-CSIC), and the commonly identified proteins in the three biological replicates of each condition were recorded. Proteins were grouped according to their biological function, after automatically annotated using the software Blast2go (BioBam, Spain) and manually checked on the UniProt Protein Database (http://www.uniprot.org/). Groups consisted of proteins related to cytoskeleton, structural membrane component, locomotion, carbohydrate metabolism, protein and amino acid metabolism, lipid metabolism, nucleotide metabolism, energetic metabolism, oxidative metabolism, cellular stress, mitochondrial proteins, nucleus, proteases, ribosomal, signaling, miscellaneous and unidentified proteins (undefined functions). Identified proteins exclusively present in EVs, in both EVs and EVs-free supernatant or exclusively present in the EVs-free supernatant were organized depending growth condition, and are presented as Supplementary Tables 1 and 2.

### SDS-PAGE electrophoresis and zymography

Peptidases have been characterized by MS and confirmed as described previously [94]. In summary, 0.1% gelatin as substrate was incorporated into a polyacrylamide gel containing sodium dodecyl sulfate (SDS-PAGE). The equivalent to 50 μg of proteins from EVs or EVs-free supernatants from each growth condition were applied into each gel slot and samples submitted to electrophoresis at a constant current of 100 V at 4°C, until the bottom edge of the gel was reached. Gels were incubated in 2.5% Triton X-100 for 1 h at room temperature under constant agitation. The same gel set-ups were then further incubated in the following buffer conditions for 48 h at 37°C in order to determine the best pH value for peptidase activity: sodium acetate buffer 0.1 M (pH 3.0 and 5.0), PBS buffer (pH 7.0) and glycine buffer 0.1 M (pH 9.0). Gels were stained overnight with 0.2% solution of Coomassie Brilliant Blue R-250 in methanol/ acetic acid/ water (50:10:40) and destained in methanol: acetic acid: water (5:10:85). Gels were dried and had their images scanned for further digital processing.

### Confocal microscopy of CHO and T98G cell lines.

CHO and T98G and cells were plated at 10^5^ cells/well onto 24-well plates covered with sterile glass coverslips, and cultured overnight at 37ºC/5% CO_2_. EVs (20 µg) were stained with 3 mM Dil (1,1'-Dioctadecyl-3,3,3', 3'-Tetramethylindocarbocyanine Perchlorate; DiIC18, Life Technologies) at 4^0^C for 30 min. EVs were ultra-centrifuged at 100,000 *xg* for 1 h and the were re-suspended in 3 mL of 0.22 µm filtered PBS, and washing repeated three more times. EVs were added to both CHO and T98G cells and incubated for different intervals (5, 10, 15, 30, 45 and 60 min). After the incubations, wells were washed will PBS and fixed with 4% paraformaldehyde for 40 min at 37^0^C. Cells were washed and blocked with 1% PBS-BSA. Cells were washed and labelled with Cholera Toxin Subunit B (CtxB, Recombinant)- Alexa Fluor 488 (Sigma-Aldrich) at 1 µg/ml for 1 h at 4^0^C. After three more washes, cells were labeled with 4,6-diamidino-2-phenylindole (DAPI) (10 µgml−1) for 30 min at room temperature. The coverslips were washed three times with PBS and mounted in 50% glycerol and 50mM n-propyl gallate in PBS. The slides were visualized in a Confocal Microscope Zeiss LSM 710NLO-Meta with 40x objective DAPI-labelled cell nuclei was excited with a two-photon excitation regime using a Mai Tai HPpulsed infrared laser (Spectra-Physics, Lasers) at 740 nm. CtxB-Alexa 488 and DilC18-labelled EVs were excited with argon ion laser at 488 nm and a diode laser at 561 nm, respectively. Emissions were collected in thee separated channels using bandpass filters for green (BP 500–550 IR), red (BP 575–615 IR) and blue (BP 435–485 IR) channels. All images were collected using the AxioVision 4.8. software (Carl Zeiss). Pinhole diameters were set to 1 Airy unit corresponding to a z resolution of 0.8 μm. Displayed results are representative of three independent experiments.

### MTT cell culture viability

CHO (hamster ovarium) and T98G cells were plated on a 96-wels plate (2 × 10^5^ cells/well) and cultured overnight at 37ºC/5% CO_2_. Wells were washed with medium in the absence of serum. Serial dilutions (from 20 to 0.010 µg/mL) of either EVs or EVs-free supernatants in serum-supplemented medium were added to cells. As controls, CHO or T98 were also incubated with irrelevant vesicle extracted from J774.16 macrophage-like cells, PYG medium or BSA at distinct proteins concentrations. Additionally, EVs and EVs-free supernatant were added in the presence of protease inhibitors phenylmethylsulfonyl fluoride (PMSF), phenantroline, pepstatin A, trans-Epoxysuccinyl-L-leucylamido(4-guanidino)butane (E-64) and the complete mini protease inhibitors cocktail (Sigma-Aldrich) following the manufacture's recomended concentrations. Plates were incubated overnight at 37ºC/5% CO_2_, washed with PBS, and then cell viability was assessed by the Methylthiazolyldiphenyl- tetrazolium bromide (MTT) assay [95]. Experiments were performed in duplicates with the three biological replicates of each EVs or EV-free supernatant from the distinct growth condition.

### Annexin/PI apoptosis assay

Using an 8-well culture chamber (Lab-Tek®), CHO or T98G were plated at 5 × 10^5^ cells/well and cultured overnight at 37ºC/5% CO_2_. Wells were washed with medium in the absence of serum and 20 µg/mL of either EVs or EVs-free supernatant were added to the cells for 6 hours. Saponin has been used as positive control for cell death [96], and for this approach, both cell lines were treated with 100 mM of saponin for 15 min at 4°C [97]. After this period, cells were fixed with 4% paraformaldehyde for 30 min at RT, and then washed with sterile PBS. For the apoptosis assay, 10 µL of the ligand buffer and 90 µL of sterile PBS were added to 8 µL of the propidium iodide (PI) solution and 8 µL Annexin V-FITC conjugated (Annexin V-FITC Apoptosis Detection Kit – SIGMA) and chambers incubated for 15 min at RT in the absence of light. This combination allows the differentitation of viable cells (PI^−^, annexin V^−^), apoptotic cells (PI^−^, annexin V^+^) and necrotic cells (PI^+^, annexin V^+^). The slides were then prepared for visualization in the Zeiss Axiovert 200 fluorescence inverted phase and contrast microscopy using a 100X objective (Carl Zeiss MicroImaging, Inc.). Annexin V-FITC and PI labeled cells were detected by the GFP and TRITC filters, respectively and fluorescences collected separated in two channels. DIC and fluorescence microscopy pictures were recorded. Images were analyzed by the Axiovision (LE) according to manufacturer's instructions and Image J (NIH, Bethesda) as described [98,99]. Cells in each picture were individually gated and fluorescence intensitiy of each individual cells were recorded by channel. At least 500 cells were analyzed for each condition. Fluorescence intensity for Annexin V-FITC and PI were plotted for each cell. Displayed results are representative of three independent experiments.

### Statistical analysis

Unpaired Student T-test or Spearman correlations were performed using Prism version 7.00 for Windows, GraphPad Software (La Jolla California USA, www.graphpad.com).

## Supplementary Material

1451184.zip
