# Supplementary material for: Extracellular vesicles and vesicle-free secretome of the protozoa Acanthamoeba castellanii under homeostasis and nutritional stress and their damaging potential to host cells
Source: Virulence. 2018 May 4;9(1):818–36. doi: 10.1080/21505594.2018.1451184 (PMC5955443; doi:10.1080/21505594.2018.1451184)
Supplement: 1451184.zip [file kvir-09-01-1451184-s001.zip › 1451184/Supplementary table 1.docx]

| **PYG-EVs exclusive proteins** | | | | | |
| --- | --- | --- | --- | --- | --- |
| **Uniprot Accession number** | | | **Protein Name** | **Protein Class** | **Molecular weight (Da)** |
| L8GSY8 | | | Uncharacterized protein | Cytoskeleton | 131,967 |
| L8GVM5 | | | Talin | Cytoskeleton | 120,263 |
| L8H0N8 | | | Actin subfamily protein | Cytoskeleton | 31,110 |
| L8HCZ7 | | | Actin-1 | Cytoskeleton | 41,676 |
| L8HDK5 | | | Actin related protein 3 | Cytoskeleton | 47,983 |
| L8HET7 | | | Ph domain containing protein | Cytoskeleton | 52,619 |
| L8HGQ4 | | | Actin related protein 2 | Cytoskeleton | 44,229 |
| L8HJ59 | | | Factin-capping protein subunit beta | Cytoskeleton | 31,162 |
| L8HJA5 | | | Actin related protein 2 isoform 4 | Cytoskeleton | 44,192 |
| L8GE59 | | | Plasma membrane ATPase | Structural membrane component | 112,656 |
| L8GJY4 | | | ABC transporter, ATPbinding domain containing protein | Structural membrane component | 69,338 |
| L8H1N2 | | | Filamin repeat domain containing protein | Structural membrane component | 88,770 |
| L8HCE4 | | | IRSp53/MIM domain containing protein | Structural membrane component | 39,784 |
| L8H1F4 | | | Myosin VIIa | Locomotion | 232,886 |
| L8HHR2 | | | Myosin IC heavy chain | Locomotion | 125,006 |
| L8HMU1 | | | Myosin2 heavy chain | Locomotion | 171,216 |
| L8GML0 | | | Myosin-light-chain kinase | Protein and amino acid metabolism | 14,593 |
| L8GP31 | | | Serine/threonine kinase 24 | Protein and amino acid metabolism | 50,617 |
| L8GUU1 | | | Serine/threonine kinase | Protein and amino acid metabolism | 39,217 |
| L8GWQ5 | | | Protein kinase domain containing protein | Protein and amino acid metabolism | 148,656 |
| L8GY18 | | | Protein kinase domain containing protein | Protein and amino acid metabolism | 111,296 |
| L8GYD3 | | | Uncharacterized protein | Protein and amino acid metabolism | 40,396 |
| L8H8C6 | | | Protein kinase domain containing protein | Protein and amino acid metabolism | 52,031 |
| R4L876 | | | Methionine synthase | Protein and amino acid metabolism | 90,503 |
| L8GHW5 | | | C8 sterol isomerase | Lipid metabolism | 21,546 |
| L8GVZ1 | | | LBP / BPI / CETP family, Cterminal domain containing protein | Lipid metabolism | 52,290 |
| L8H3W2 | | | Lipid transport family protein | Lipid metabolism | 89,696 |
| L8H655 | | | Phospholipase | Lipid metabolism | 47,904 |
| L8GEM3 | | | ABC transporter A family protein | Energetic metabolism | 73,327 |
| L8GVQ1 | | | Vacuolar proton ATPase, putative | Energetic metabolism | 25,615 |
| L8H1B3 | | | Non-specific protein-tyrosine kinase | Energetic metabolism | 51,217 |
| L8H6F0 | | | ABC2 type transporter superfamily protein | Energetic metabolism | 78,703 |
| L8GH71 | | | Protein disulfide-isomerase | Oxidative metabolism | 52,926 |
| L8H6T6 | | | Heat shock protein 83 | Cellular stress | 46,263 |
| L8GMR7 | | | Eukaryotic porin protein | Mitochondrial proteins | 33,586 |
| L8GUU6 | | | Adenine nucleotide translocator | Mitochondrial proteins | 34,021 |
| L8GXI3 | | | Rho family, small GTP binding protein Rac3 | Nucleus | 21,686 |
| L8H192 | | | Actin-related protein 2/3 complex subunit 5 | Nucleus | 13,950 |
| L8HG55 | | | Ras subfamily protein | Nucleus | 18,012 |
| L8HGB4 | | | RabE family small GTPase | Nucleus | 17,380 |
| L8GJ32 | | | 40s ribosomal protein s4 | Ribosomal | 27,273 |
| L8GNH8 | | | Rpl7A | Ribosomal | 31,732 |
| L8GPQ3 | | | Ribosomal protein L10 | Ribosomal | 34,791 |
| L8GQT3 | | | 40S ribosomal protein S3a | Ribosomal | 29,244 |
| L8GRL5 | | | Ribosomal protein L23 | Ribosomal | 23,234 |
| L8GVI2 | | | 60S ribosomal protein L13 | Ribosomal | 24,417 |
| L8GX53 | | | 60S ribosomal protein | Ribosomal | 12,366 |
| L8GXK0 | | | Ribosomal protein L32 | Ribosomal | 15,714 |
| L8GY75 | | | Ribosomal protein L6e | Ribosomal | 27,470 |
| L8GZ94 | | | Ribosomal L5P family Cterminus | Ribosomal | 12,523 |
| L8H1X4 | | | Ribosomal protein L3 | Ribosomal | 45,111 |
| L8H2L6 | | | Ribosomal protein L22 | Ribosomal | 19,867 |
| L8H2M8 | | | Ribosomal protein S9 | Ribosomal | 16,561 |
| L8H6U1 | | | 60s Acidic ribosomal protein | Ribosomal | 12,306 |
| L8HAU5 | | | Ribosomal protein S13p/S18e | Ribosomal | 18,934 |
| L8HF06 | | | S25 ribosomal protein | Ribosomal | 14,981 |
| L8HGC7 | | | 60S ribosomal protein L27a | Ribosomal | 20,276 |
| L8HHU3 | | | Ribosomal protein S15 | Ribosomal | 16,941 |
| L8HIT8 | | | Ribosomal protein L4/L1 family | Ribosomal | 43,793 |
| L8HJ08 | | | 40S ribosomal protein S8 | Ribosomal | 27,825 |
| L8HJG1 | | | Ribosomal protein L19 | Ribosomal | 34,158 |
| L8HM38 | | | Eukaryotic ribosomal protein L18 | Ribosomal | 20,903 |
| L8GR43 | | | RAP1A, member of RAS oncogene family | Signaling | 22,280 |
| L8H896 | | | RAB1B, member RAS oncogene family | Signaling | 22,332 |
| L8H8P6 | | | Raslike protein 1 | Signaling | 22,559 |
| L8H8S8 | | | Ras subfamily protein | Signaling | 14,253 |
| L8HCY9 | | | Ras-related C3 botulinum toxin substrate 3 | Signaling | 16,844 |
| L8HD31 | | | Rasrelated protein ORAB-1 | Signaling | 23,048 |
| L8HJ94 | | | Raclike protein | Signaling | 24,794 |
| L8GFE0 | | | Nodulin 21 family protein | Miscellaneous | 27,839 |
| L8GLW9 | | | Calmodulin | Miscellaneous | 16,829 |
| L8GNN8 | | | Lim domain containing protein | Miscellaneous | 18,989 |
| L8GQE7 | | | Armadillo/betacatenin-like repeat domain containing protein | Miscellaneous | 54,138 |
| L8GQU5 | | | Major Vault Protein repeatcontaining protein | Miscellaneous | 86,221 |
| L8H8H2 | | | Calmodulin | Miscellaneous | 7,481 |
| L8HAN0 | | | Uncharacterized protein | Miscellaneous | 44,666 |
| L8HCY6 | | | EF hand domain containing protein | Miscellaneous | 16,054 |
| L8HHV3 | | | Syntaxin protein | Miscellaneous | 34,388 |
| L8HKQ5 | | | Amidohydrolase domain containing protein | Miscellaneous | 123,298 |
| L8GI20 | | | Uncharacterized protein | Unidentified protein | 18,530 |
| L8GKR8 | | | Uncharacterized protein | Unidentified protein | 95,336 |
| L8GM07 | | | Uncharacterized protein | Unidentified protein | 20,556 |
| L8GRC6 | | | Uncharacterized protein | Unidentified protein | 17,920 |
| L8GVU6 | | | Uncharacterized protein | Unidentified protein | 20,067 |
| L8GWC9 | | | Uncharacterized protein | Unidentified protein | 41,242 |
| L8GXE8 | | | Uncharacterized protein | Unidentified protein | 33,152 |
| L8H4N4 | | | Uncharacterized protein | Unidentified protein | 22,477 |
| L8H6F1 | | | p53 inducible protein | Unidentified protein | 163,454 |
| L8HAX9 | | | Uncharacterized protein | Unidentified protein | 27,184 |
|  | | |  |  |  |
| **PYG-EVs and EVs-free supernatant common proteins** | | | | | |
| **Uniprot Accession number** | **Protein Name** | | | **Protein Class** | **Molecular weight (Da)** |
| L8HDD6 | Filamin repeat domain containing protein | | | Cytoskeleton | 94,065 |
| L8GX61 | Alpha-mannosidase | | | Carbohydrate metabolism | 111,557 |
| L8H5C3 | Glycosyl hydrolase family 65 central catalytic domain containing protein | | | Carbohydrate metabolism | 81,151 |
| L8HCH4 | Beta-mannosidase mannanase mannase family protein | | | Carbohydrate metabolism | 80,474 |
| L8GW94 | Uncharacterized protein | | | Lipid metabolism | 45,157 |
| L8H9L6 | Carboxylic ester hydrolase | | | Lipid metabolism | 57,686 |
| L8GG65 | Inosineuridine preferring nucleoside hydrolase family protein | | | Nucleotide metabolism | 37,659 |
| L8H1F8 | Inosineuridine preferring nucleoside hydrolase family protein | | | Nucleotide metabolism | 33,970 |
| L8GJT1 | Peroxidase | | | Oxidative metabolism | 134,070 |
| L8H1H6 | Serine proteinase | | | Protease | 38,022 |
| L8GTD0 | Chorismate mutase subfamily protein | | | Miscellaneous | 20,759 |
| L8GTG4 | Chorismate mutase subfamily protein | | | Miscellaneous | 20,849 |
| L8H749 | Uncharacterized protein | | | Miscellaneous | 62,994 |
| L8HDT6 | Chorismate mutase | | | Miscellaneous | 20,869 |
| L8GHM4 | Uncharacterized protein | | | Unidentified protein | 12,778 |
| L8GRX4 | Uncharacterized protein | | | Unidentified protein | 21,176 |
| L8GUI9 | Uncharacterized protein | | | Unidentified protein | 19,167 |
| L8GUQ6 | Uncharacterized protein | | | Unidentified protein | 33,579 |
| L8GV29 | Uncharacterized protein | | | Unidentified protein | 19,514 |
| L8H7Z5 | Uncharacterized protein | | | Unidentified protein | 73,533 |
| L8HGF5 | Uncharacterized protein | | | Unidentified protein | 19,064 |
|  |  | | |  |  |
| **PYG-EVs-free supernatant exclusive proteins** | | | | | |
| **Uniprot Accession number** | | **Protein Name** | | **Protein Class** | **Molecular weight (Da)** |
| L8H3N1 | | gelation factor | | Cytoskeleton | 80,157 |
| L8GT58 | | Fasciclin domain containing protein | | Structural membrane component | 53,300 |
| L8H4F3 | | alkaline phosphatase | | Structural membrane component | 62,306 |
| L8GH69 | | Glycosyl hydrolase family 20, catalytic domain containing protein | | Carbohydrate metabolism | 39,794 |
| L8GLE7 | | Glycosyl hydrolase | | Carbohydrate metabolism | 108,173 |
| L8GRI1 | | Glycosyl hydrolase family 3, C-terminal domain containing protein | | Carbohydrate metabolism | 22,493 |
| L8GRZ9 | | Enolase, Cterminal TIM barrel domain containing protein | | Carbohydrate metabolism | 46,589 |
| L8GUL5 | | Glycosyl hydrolase | | Carbohydrate metabolism | 48,406 |
| L8GX26 | | Betaglucosidase | | Carbohydrate metabolism | 42,412 |
| L8GZ68 | | Xylosidase | | Carbohydrate metabolism | 90,404 |
| L8H5H5 | | Glycoside hydrolase family protein | | Carbohydrate metabolism | 57,743 |
| L8H5T2 | | Uncharacterized protein | | Carbohydrate metabolism | 47,961 |
| L8H838 | | Beta-galactosidase | | Carbohydrate metabolism | 67,006 |
| L8HGN3 | | Uncharacterized protein | | Carbohydrate metabolism | 15,556 |
| L8HI17 | | Alpha amylase, catalytic subfamily protein | | Carbohydrate metabolism | 60,735 |
| L8HIM5 | | Acidstable alpha-amylase | | Carbohydrate metabolism | 53,108 |
| L8H2V5 | | Hydrolase, alpha/beta fold domain containing protein | | Protein and amino acid metabolism | 93,344 |
| L8HJ93 | | 4-hydroxyphenylpyruvate dioxygenase | | Protein and amino acid metabolism | 46,216 |
| L8GEL0 | | Carboxylesterase superfamily protein | | Lipid metabolism | 60,424 |
| L8GV58 | | Acetylcholinesterase | | Lipid metabolism | 27,873 |
| L8GZN3 | | Carboxylic ester hydrolase | | Lipid metabolism | 34,006 |
| L8H8T7 | | Pectinacetylesterase | | Lipid metabolism | 47,880 |
| L8GZK2 | | Phospholipase B-like | | Lipid metabolism | 53,430 |
| L8GQ21 | | Uricase | | Nucleotide metabolism | 33,423 |
| L8GQ41 | | Inosineuridine preferring nucleoside hydrolase family protein | | Nucleotide metabolism | 42,486 |
| L8GQE5 | | Nucleoside diphosphate kinase | | Nucleotide metabolism | 25,432 |
| L8H3N0 | | Protein kinase domain containing protein | | Nucleotide metabolism | 161,517 |
| L8H0Z8 | | Thiazole biosynthesis protein ThiG | | Energetic metabolism | 29,867 |
| L8GFU3 | | Uncharacterized protein | | Oxidative metabolism | 49,483 |
| L8GSI3 | | FAD binding domain containing protein | | Oxidative metabolism | 52,342 |
| L8GU40 | | Uncharacterized protein | | Oxidative metabolism | 71,449 |
| L8GWR1 | | Uncharacterized protein | | Oxidative metabolism | 14,916 |
| L8HAH1 | | Citrate synthase | | Mitochondrial proteins | 55,033 |
| L8GD90 | | Serine carboxypeptidase s28 protein | | Protease | 63,308 |
| L8GFY8 | | Carboxypeptidase A1 | | Protease | 47,540 |
| L8GI82 | | Peptidase family M13 | | Protease | 79,879 |
| L8GSQ9 | | Peptidase S8 and S53 subtilisin kexin sedolisin | | Protease | 44,359 |
| L8GZN2 | | PA domain containing protein | | Protease | 58,818 |
| L8H5Z5 | | Dipeptidyl-peptidase family protein | | Protease | 54,723 |
| L8H7U8 | | Alanyl dipeptidyl peptidase | | Protease | 78,270 |
| L8HJ51 | | Aspartic proteinase | | Protease | 52,867 |
| L8HLI8 | | Cysteine proteinase | | Protease | 36,208 |
| L8HLJ4 | | Carboxypeptidase A3 | | Protease | 46,979 |
| L8HMP8 | | Serine carboxypeptidase S28 | | Protease | 59,851 |
| L8GPC9 | | LmbE family protein | | Signaling | 30,549 |
| L8HAQ5 | | AlphaN-acetylglucosaminidase (NAGLU) | | Signaling | 90,923 |
| L8HJB2 | | Calponin domain containing protein | | Signaling | 100,844 |
| L8GCL7 | | Uncharacterized protein | | Miscellaneous | 64,274 |
| L8GF26 | | Uncharacterized protein | | Miscellaneous | 46,026 |
| L8GIC1 | | NlpC/P60 domain containing protein | | Miscellaneous | 34,660 |
| L8GPR8 | | Uncharacterized protein | | Miscellaneous | 78,477 |
| L8GPZ0 | | Laminin egflike (Domains iii and v) domain containing protein | | Miscellaneous | 193,143 |
| L8GRQ1 | | Uncharacterized protein | | Miscellaneous | 46,854 |
| L8GUY8 | | Communication mutant protein f | | Miscellaneous | 129,358 |
| L8GXW3 | | Zinc finger protein | | Miscellaneous | 52,274 |
| L8GZQ0 | | Prepeptidase C-terminal domain containing protein | | Miscellaneous | 22,852 |
| L8H373 | | Uncharacterized protein | | Miscellaneous | 36,290 |
| L8H5L5 | | Histidine acid phosphatase superfamily protein | | Miscellaneous | 55,143 |
| L8HC86 | | Uncharacterized protein | | Miscellaneous | 61,450 |
| L8GFH6 | | Uncharacterized protein | | Unidentified protein | 24,675 |
| L8GG92 | | Uncharacterized protein | | Unidentified protein | 25,053 |
| L8GGC1 | | Uncharacterized protein | | Unidentified protein | 39,267 |
| L8GLY8 | | Uncharacterized protein | | Unidentified protein | 42,252 |
| L8GUF7 | | Uncharacterized protein | | Unidentified protein | 41,489 |
| L8GX90 | | Uncharacterized protein | | Unidentified protein | 18,725 |
| L8H071 | | Zinc finger protein | | Unidentified protein | 53,342 |
| L8H3E5 | | Uncharacterized protein | | Unidentified protein | 70,725 |
| L8HJI3 | | Uncharacterized protein | | Unidentified protein | 20,562 |
| L8HK96 | | Uncharacterized protein | | Unidentified protein | 18,951 |
