# Supplementary material for: Extracellular vesicles and vesicle-free secretome of the protozoa Acanthamoeba castellanii under homeostasis and nutritional stress and their damaging potential to host cells
Source: Virulence. 2018 May 4;9(1):818–36. doi: 10.1080/21505594.2018.1451184 (PMC5955443; doi:10.1080/21505594.2018.1451184)
Supplement: 1451184.zip [file kvir-09-01-1451184-s001.zip › 1451184/Supplementary table 2.docx]

| **Glucose-EVs exclusive proteins** | | | |
| --- | --- | --- | --- |
| **Uniprot Accession number** | **Protein Name** | **Protein Class** | **Molecular weight (Da)** |
| L8GG08 | Actin bundling protein | Cytoskeleton | 31,046 |
| L8GSY8 | Uncharacterized protein | Cytoskeleton | 131,967 |
| L8GWZ2 | ARP2/3 complex 34 kDa subunit | Cytoskeleton | 33,355 |
| L8GZW8 | Actinbinding protein fragmin P | Cytoskeleton | 20,568 |
| L8HMG4 | Adenylyl cyclase-associated protein | Cytoskeleton | 51,564 |
| L8HCZ7 | Actin-1 | Cytoskeleton | 41,676 |
| L8HDC1 | Actin-related protein 2/3 complex subunit 3 | Cytoskeleton | 18,060 |
| L8HDK5 | Actin related protein 3 | Cytoskeleton | 47,983 |
| L8GE59 | Plasma membrane ATPase | Structural membrane component | 112,656 |
| L8GT58 | Fasciclin domain containing protein | Structural membrane component | 53,300 |
| L8GVH2 | Phospholipid-transporting ATPase | Structural membrane component | 129,759 |
| L8H4F3 | Alkaline phosphatase | Structural membrane component | 62,306 |
| L8HMU1 | Myosin2 heavy chain | Locomotion | 171,216 |
| L8GWF8 | Phosphoenolpyruvate carboxylase | Carbohydrate metabolism | 103,464 |
| L8H880 | Glycosyl hydrolases family 25 subfamily protein | Carbohydrate metabolism | 70,775 |
| L8H8A5 | Alpha-1,4 glucan phosphorylase | Carbohydrate metabolism | 101,490 |
| L8GHJ0 | Sortilin | Protein and amino acid metabolism | 84,475 |
| L8GIG1 | Tryptophan-tRNA ligase | Protein and amino acid metabolism | 45,868 |
| R4L876 | Methionine synthase | Protein and amino acid metabolism | 90,503 |
| L8GMK5 | Gammaadaptin 1 | Protein and amino acid metabolism | 84,121 |
| L8H0W1 | L-glutamine synthetase | Protein and amino acid metabolism | 34,759 |
| L8H146 | Eukaryotic initiation factor | Protein and amino acid metabolism | 33,315 |
| L8H1T2 | Carbamoylphosphate synthase, large subunit | Protein and amino acid metabolism | 248,448 |
| L8H265 | Vacuolar sorting protein | Protein and amino acid metabolism | 93,499 |
| L8H280 | Glutamine synthetase | Protein and amino acid metabolism | 51,875 |
| L8H2W6 | Golgi family protein | Protein and amino acid metabolism | 42,567 |
| L8H3E1 | Eukaryotic translation initiation factor 3 subunit B | Protein and amino acid metabolism | 79,427 |
| L8H6J0 | Isoleucyl-tRNA synthetase | Protein and amino acid metabolism | 95,052 |
| L8H7Q1 | Histidine ammonia-lyase | Protein and amino acid metabolism | 58,526 |
| L8H955 | Eukaryotic translation initiation factor 2 | Protein and amino acid metabolism | 52,753 |
| L8H9E4 | Probable methylthioribulose-1-phosphate dehydratase | Protein and amino acid metabolism | 24,289 |
| L8HLD9 | Ureidopropionase, beta | Protein and amino acid metabolism | 41,392 |
| L8GVZ1 | LBP / BPI / CETP family, Cterminal domain containing protein | Lipid metabolism | 52,290 |
| L8GW94 | Uncharacterized protein | Lipid metabolism | 45,157 |
| L8GXB0 | ATP-citrate synthase | Lipid metabolism | 123,889 |
| L8GZJ4 | Acyl-coenzyme A oxidase | Lipid metabolism | 83,479 |
| L8H8H3 | Carboxylic ester hydrolase | Lipid metabolism | 55,496 |
| L8HAK4 | 3-hydroxyacyl-CoA dehydrogenase | Lipid metabolism | 34,917 |
| L8HG13 | Choline/Carnitine oacyltransferase superfamily protein | Lipid metabolism | 124,859 |
| L8GG65 | Inosineuridine preferring nucleoside hydrolase family protein | Nucleotide metabolism | 37,659 |
| L8GQE5 | Nucleoside diphosphate kinase | Nucleotide metabolism | 25,432 |
| L8H104 | CBS domain containing protein | Nucleotide metabolism | 34,787 |
| L8HGD4 | Inosine5'-monophosphate dehydrogenase | Nucleotide metabolism | 59,163 |
| L8H4W8 | Oxoglutarate dehydrogenase (Succinyltransferring), E1 component | Energetic metabolism | 114,420 |
| L8HCS5 | Acetyltransferase component of pyruvate dehydrogenase complex | Energetic metabolism | 53,012 |
| L8HHZ1 | V-type proton ATPase subunit a | Energetic metabolism | 92,623 |
| L8GI68 | Antibiotic biosynthesis monooxygenase subfamily protein | Oxidative metabolism | 13,874 |
| L8GJT1 | Peroxidase | Oxidative metabolism | 134,070 |
| L8GK33 | Amine oxidase, flavincontaining superfamily protein | Oxidative metabolism | 76,094 |
| L8GRJ6 | Calponin domain containing protein | Oxidative metabolism | 46,989 |
| L8GSI3 | FAD binding domain containing protein | Oxidative metabolism | 52,342 |
| L8GV93 | Shortchain dehydrogenase/reductase SDR | Oxidative metabolism | 33,055 |
| L8HCW6 | PPOD1 peroxidase | Oxidative metabolism | 14,055 |
| L8HDW9 | Oxidoreductase, zincbinding dehydrogenase | Oxidative metabolism | 40,383 |
| L8HFV6 | Universal stress domain containing protein | Cellular stress | 22,956 |
| L8GMR7 | Eukaryotic porin protein | Mitochondrial proteins | 33,586 |
| L8H192 | Actin-related protein 2/3 complex subunit 5 | Nucleus | 13,950 |
| L8H4F8 | Histone H2B | Nucleus | 17,322 |
| L8GPK5 | Aspartyl aminopeptidase | Protease | 15,170 |
| L8GTW2 | Peptidase M16 family protein | Protease | 53,866 |
| L8HEC6 | Peptidase, S8/S53 subfamily protein | Protease | 137,641 |
| L8GFS4 | Ribosomal protein S5 | Ribosomal | 27,130 |
| L8GJ32 | 40s ribosomal protein s4 | Ribosomal | 27,273 |
| L8GJF7 | Ribosomal protein L6 | Ribosomal | 21,447 |
| L8GJX8 | Ribosomal L40e family | Ribosomal | 14,684 |
| L8GNH8 | Rpl7A | Ribosomal | 31,732 |
| L8GPQ3 | Ribosomal protein L10 | Ribosomal | 34,791 |
| L8GQT3 | 40S ribosomal protein S3a | Ribosomal | 29,244 |
| L8GY75 | Ribosomal protein L6e | Ribosomal | 27,470 |
| L8GYM5 | Ribosomal protein L15 | Ribosomal | 23,028 |
| L8H6U1 | 60s Acidic ribosomal protein | Ribosomal | 12,306 |
| L8H9Q4 | Ribosomal protein s27a | Ribosomal | 17,847 |
| L8HA34 | Ribosomal protein S17 | Ribosomal | 19,099 |
| L8HC77 | Ribosomal protein S7p/S5e | Ribosomal | 22,225 |
| L8HCV0 | Ribosomal protein L13 | Ribosomal | 23,316 |
| L8HD98 | Ribosomal protein S19 | Ribosomal | 12,549 |
| L8HEU0 | Ribosomal protein S7e | Ribosomal | 21,930 |
| L8HGC7 | 60S ribosomal protein L27a | Ribosomal | 20,276 |
| L8HHU3 | Ribosomal protein S15 | Ribosomal | 16,941 |
| L8HIT8 | Ribosomal protein L4/L1 family | Ribosomal | 43,793 |
| L8HJ84 | Ribosomal protein L31e | Ribosomal | 13,898 |
| L8HJG1 | Ribosomal protein L19 | Ribosomal | 34,158 |
| L8HM38 | Eukaryotic ribosomal protein L18 | Ribosomal | 20,903 |
| L8GR43 | RAP1A, member of RAS oncogene family | Signaling | 22,280 |
| L8H6I9 | SH3 domain containing protein | Signaling | 56,923 |
| L8H8P6 | Raslike protein 1 | Signaling | 22,559 |
| L8GDL8 | Ubiquitin domain containing protein | Miscellaneous | 25,190 |
| L8GEU6 | 26S proteasome nonATPase regulatory subunit 7 | Miscellaneous | 36,820 |
| L8GF26 | Uncharacterized protein | Miscellaneous | 46,026 |
| L8GF48 | CBS domain containing protein | Miscellaneous | 30,099 |
| L8GH21 | Von Willebrand factor type A domain containing protein | Miscellaneous | 22,497 |
| L8GHR5 | Von willebrand factor type a domain containing protein | Miscellaneous | 36,944 |
| L8GI05 | Von willebrand factor type a domain containing protein | Miscellaneous | 39,058 |
| L8GJT5 | Polyubiquitin | Miscellaneous | 24,854 |
| L8GPZ0 | Laminin egflike (Domains iii and v) domain containing protein | Miscellaneous | 193,143 |
| L8GQ68 | SH3 domain containing protein | Miscellaneous | 52,486 |
| L8GQ98 | Uncharacterized protein | Miscellaneous | 27,965 |
| L8GQU5 | Major Vault Protein repeatcontaining protein | Miscellaneous | 86,221 |
| L8GRB9 | MyosinI binding protein | Miscellaneous | 117,938 |
| L8GT53 | Sec1like family protein | Miscellaneous | 68,954 |
| L8GV78 | Ornithine/lysine/arginine decarboxylase | Miscellaneous | 112,534 |
| L8GWH1 | Polyubiquitin | Miscellaneous | 24,854 |
| L8GXA8 | Copine VIII | Miscellaneous | 64,292 |
| L8GY68 | Glia maturation factor family protein | Miscellaneous | 16,852 |
| L8GYR7 | Proteasome subunit alpha type | Miscellaneous | 25,198 |
| L8GZK1 | Elongation factor 1alpha, somatic form | Miscellaneous | 35,896 |
| L8H0Y2 | Polyubiquitin | Miscellaneous | 25,866 |
| L8H122 | Vacuolar proton pump d subunit | Miscellaneous | 35,205 |
| L8H2D5 | AMPbinding enzyme domain containing protein | Miscellaneous | 49,186 |
| L8H8R6 | Vacuolar protein sortingassociated protein 26 | Miscellaneous | 39,416 |
| L8HAW7 | Autophagy-related protein 27 | Miscellaneous | 28,844 |
| L8HBA5 | Ubiquitin domain containing protein | Miscellaneous | 116,575 |
| L8HC65 | C2 domain containing protein | Miscellaneous | 17,301 |
| L8HCL5 | Ubiquitinfusion protein | Miscellaneous | 15,952 |
| L8HED5 | C2 domain containing protein | Miscellaneous | 14,171 |
| L8HIC0 | 20S proteasome subunit beta 3 | Miscellaneous | 21,474 |
| L8HKL1 | PH domain containing protein | Miscellaneous | 45,641 |
| L8GEC2 | Uncharacterized protein | Unidentified protein | 133,037 |
| L8GKH0 | Uncharacterized protein | Unidentified protein | 64,137 |
| L8GKR8 | Uncharacterized protein | Unidentified protein | 95,336 |
| L8GN71 | HEAT repeat domain containing protein | Unidentified protein | 178,204 |
| L8GTS0 | Uncharacterized protein | Unidentified protein | 22,007 |
| L8GUQ6 | Uncharacterized protein | Unidentified protein | 33,579 |
| L8GV29 | Uncharacterized protein | Unidentified protein | 19,514 |
| L8GWC9 | Uncharacterized protein | Unidentified protein | 41,242 |
| L8GWT1 | TolA | Unidentified protein | 46,670 |
| L8GXC9 | Uncharacterized protein | Unidentified protein | 52,777 |
| L8H326 | Uncharacterized protein | Unidentified protein | 22,154 |
| L8H4K6 | TolAlike protein | Unidentified protein | 35,606 |
| L8HEL3 | Uncharacterized protein | Unidentified protein | 44,445 |
| L8HFE5 | Uncharacterized protein | Unidentified protein | 8,444 |
| L8HGZ6 | Uncharacterized protein | Unidentified protein | 42,999 |
| L8HKF5 | Uncharacterized protein | Unidentified protein | 25,920 |
| L8HM01 | Uncharacterized protein | Unidentified protein | 57,527 |
|  |  |  |  |
| **Glucose-EVs and EVs-free supernatant common proteins** | | | |
| **Uniprot Accession number** | **Protein Name** | **Protein Class** | **Molecular weight (Da)** |
| L8GJG4 | Plastin 3 (T isoform) | Cytoskeleton | 57,540 |
| L8H3N1 | Gelation factor | Cytoskeleton | 80,157 |
| L8HJ75 | Alpha amylase | Carbohydrate metabolism | 60,226 |
| L8GUV2 | Chaperonin GroL | Protein and amino acid metabolism | 60,850 |
| L8HH66 | VATPase subunit A | Energetic metabolism | 64,528 |
| L8GTN6 | Catalase | Oxidative metabolism | 55,347 |
| L8H5G5 | 20s proteasome subunit c2 | Nucleus | 27,658 |
| L8HJ51 | Aspartic proteinase | Protease | 52,867 |
| L8H0P3 | 40s ribosomal protein s12 | Ribosomal | 16,613 |
| L8GT20 | Eukaryotic translation elongation factor 2 | Miscellaneous | 93,342 |
| L8H2Q1 | Elongation factor 1-gamma family protein | Miscellaneous | 46,307 |
| L8H4P8 | Guanine nucleotide-binding protein beta subunit | Miscellaneous | 35,215 |
| L8HG01 | Proteasome subunit beta type | Miscellaneous | 23,297 |
| L8H2F2 | Protein tolA | Unidentified protein | 41,852 |
|  |  |  |  |
| **Glucose-EVs-free supernatant exclusive proteins** | | | |
| **Uniprot Accession number** | **Protein Name** | **Protein Class** | **Molecular weight (Da)** |
| L8GVM5 | Talin | Cytoskeleton | 120,263 |
| L8HBF6 | Profilin | Cytoskeleton | 13,084 |
| L8HBM2 | Profilin | Cytoskeleton | 13,431 |
| L8HDD6 | Filamin repeat domain containing protein | Structural membrane component | 94,065 |
| L8GE90 | Triosephosphate isomerase | Carbohydrate metabolism | 22,447 |
| L8GHN1 | Uncharacterized protein | Carbohydrate metabolism | 34,110 |
| L8GN92 | Phosphoenolpyruvate carboxykinase (GTP) | Carbohydrate metabolism | 68,007 |
| L8GQL5 | Phosphoenolpyruvate carboxykinase (GTP), putative | Carbohydrate metabolism | 135,407 |
| L8GQP0 | Malate dehydrogenase | Carbohydrate metabolism | 33,245 |
| L8GRA3 | Glucose-6-phosphate isomerase | Carbohydrate metabolism | 63,361 |
| L8GRZ9 | Enolase, Cterminal TIM barrel domain containing protein | Carbohydrate metabolism | 46,589 |
| L8GSM5 | Serine rich protein | Carbohydrate metabolism | 30,164 |
| L8H436 | Transaldolase | Carbohydrate metabolism | 35,272 |
| L8H5H5 | Glycoside hydrolase family protein | Carbohydrate metabolism | 57,743 |
| L8H905 | Glycosyl hydrolase | Carbohydrate metabolism | 42,174 |
| L8HFT9 | Malate dehydrogenase | Carbohydrate metabolism | 45,091 |
| L8HGK3 | Glucan (1,4alpha-), branching enzyme 1 | Carbohydrate metabolism | 80,283 |
| L8HI17 | Alpha amylase, catalytic subfamily protein | Carbohydrate metabolism | 60,735 |
| L8GQ83 | Homogentisate 1,2dioxygenase | Protein and amino acid metabolism | 49,795 |
| L8GTG7 | Aspartate aminotransferase | Protein and amino acid metabolism | 46,042 |
| L8H2V5 | Hydrolase, alpha/beta fold domain containing protein | Protein and amino acid metabolism | 93,344 |
| L8HDT1 | Serine hydroxymethyltransferase | Protein and amino acid metabolism | 50,349 |
| L8HJ93 | 4-hydroxyphenylpyruvate dioxygenase | Protein and amino acid metabolism | 46,216 |
| L8HKA6 | Aspartate aminotransferase | Protein and amino acid metabolism | 48,206 |
| L8HB67 | Acid phosphatase PHOa | Lipid metabolism | 33,055 |
| L8GQ21 | Uricase | Nucleotide metabolism | 33,423 |
| L8GTE1 | Isocitrate dehydrogenase | Energetic metabolism | 45,909 |
| L8H0Z8 | Thiazole biosynthesis protein ThiG | Energetic metabolism | 29,867 |
| L8GDC4 | Superoxide dismutase | Oxidative metabolism | 16,319 |
| L8GF83 | Peroxiredoxin 2 | Oxidative metabolism | 22,119 |
| L8GGD8 | Aconitate hydratase | Oxidative metabolism | 54,427 |
| L8GZB8 | Saccharopine dehydrogenase | Oxidative metabolism | 50,219 |
| L8H3N8 | Superoxide dismutase | Oxidative metabolism | 25,037 |
| L8HET6 | Dihydrolipoyl dehydrogenase | Oxidative metabolism | 53,811 |
| L8HG59 | Thioredoxin reductase 1, cytoplasmic | Oxidative metabolism | 57,497 |
| L8GGZ4 | Heat shock protein ssa1 | Cellular stress | 68,097 |
| L8GTX7 | Succinyl-CoA:3-ketoacid-coenzyme A transferase | Mitochondrial proteins | 54,196 |
| L8GMG4 | Endonuclease | Nucleus | 33,808 |
| L8GT26 | Aminopeptidase | Protease | 93,926 |
| L8GUK2 | Tripeptidylpeptidase 1 | Protease | 57,516 |
| L8GWV6 | APM1 (AMINOPEPTIDASE M1) | Protease | 67,349 |
| L8H1H6 | Serine proteinase | Protease | 38,022 |
| L8H2I7 | Prokumamolisin, activation domain containing protein | Protease | 63,024 |
| L8HLI8 | Cysteine proteinase | Protease | 36,208 |
| L8GHD3 | Delta-aminolevulinic acid dehydratase | Miscellaneous | 36,327 |
| L8GJE3 | Cystatin | Miscellaneous | 10,543 |
| L8GNM9 | Aminotransferase | Miscellaneous | 42,474 |
| L8GU25 | Protein tyrosine phosphatase, dual specificity | Miscellaneous | 32,392 |
| L8H373 | Uncharacterized protein | Miscellaneous | 36,290 |
| L8HA89 | Farnesyl pyrophosphate synthetase | Miscellaneous | 35,404 |
| L8HH98 | Protein kinase domain containing protein | Miscellaneous | 103,703 |
| L8HJX4 | Serine/threonine-protein phosphatase | Miscellaneous | 43,241 |
| L8GFH6 | Uncharacterized protein | Unidentified protein | 24,675 |
| L8GG92 | Uncharacterized protein | Unidentified protein | 25,053 |
| L8GJ34 | Putative secreted protein | Unidentified protein | 60,855 |
| L8H385 | Uncharacterized protein | Unidentified protein | 18,874 |
| L8H7B8 | Uncharacterized protein | Unidentified protein | 25,997 |
