# Supplementary material for: Extracellular vesicles and vesicle-free secretome of the protozoa Acanthamoeba castellanii under homeostasis and nutritional stress and their damaging potential to host cells
Source: Virulence. 2018 May 4;9(1):818–36. doi: 10.1080/21505594.2018.1451184 (PMC5955443; doi:10.1080/21505594.2018.1451184)
Supplement: 1451184.zip [file kvir-09-01-1451184-s001.zip › 1451184/Supplementary table 3 new.docx]

**Supplementary Table 3.** Identified proteases by LC-MS according to their subclasses in the distinct growth conditions. Common proteins in the different fractions are shown in bold, and counted as 1 entity for the calculation of all the proteases found.

| **Protease**  **class** | **Number of identified proteases**  **(frequency-%)** | | | | | | | | | | | | **Total**  **(excluding replicates)** |
| --- | --- | --- | --- | --- | --- | --- | --- | --- | --- | --- | --- | --- | --- |
|  | **PYG-EVs** | | | **PYG-EVs-free**  **supernatant** | | | **Glucose -EVs** | | | **Glucose -EVs-free**  **supernatant** | | |  |
| Serine | 1  (100%) | **L8H1H6** | **Serine protease** | 6  (50%) | L8GD90 | Serine carboxypeptidase s28 | 1  (25%) | L8HEC6 | Peptidase, S8/S53 subfamily protein | 3  (42.9%) | L8GUK2 | Tripeptidylpeptidase 1 | 9  (47.4%) |
|  |  |  |  |  | L8GSQ9 | Peptidase S8 and S53 subtilisin kexin sedolisin |  |  |  |  |  |  |  |
|  |  |  |  |  | **L8H1H6** | **Serine protease** |  |  |  |  | **L8H1H6** | **Serine proteinase** |  |
|  |  |  |  |  | L8H5Z5 | Dipeptidyl-peptidase family protein |  |  |  |  |  |  |  |
|  |  |  |  |  | L8H7U8 | Alanyl dipeptidyl peptidase |  |  |  |  | L8H2I7 | Prokumamolisin |  |
|  |  |  |  |  | L8HMP8 | Serine carboxypeptidase S28 |  |  |  |  |  |  |  |
| Metallo | X | | | 3  (25%) | L8GFY8 | Carboxypeptidase A1 | 1  (25%) | L8GTW2 | Peptidase M16 family protein | 2  (28.6%) | L8GT26 | Uncharacterized protein | 6  (31.6%) |
|  |  |  |  |  | L8GI82 | Peptidase family M13 |  |  |  |  |  |  |  |
|  |  |  |  |  | L8HLJ4 | Carboxypeptidase A3 |  |  |  |  | L8GWV6 | APM1 (Aminopeptidase M1) |  |
| Aspartic | X | | | 1  (8.3%) | **L8HJ51** | **Aspartic proteinase** | 2  (50%) | **L8HJ51** | Aspartic proteinase | 1  (14.3%) | **L8HJ51** | **Aspartic proteinase** | 2  (10.6%) |
|  |  |  |  |  |  |  |  | L8GPK5 | Aspartyl aminopeptidase |  |  |  |  |
| Cystein | X | | | 1  (8.3%) | **L8HLI8** | **Cysteine proteinase** | X | | | 1  (14.3%) | **L8HLI8** | **Cysteine proteinase** | 1  (5.3%) |
| Not specified | X | | | 1  (8.3%) | L8GZN2 | PA domain containing protein | X | | | X | | | 1  (5.3%) |
| **Total** | 1 | | | 12 | | | 4 | | | 7 | | | 19 (100%) |
