# Supplementary figures and images for: Extracellular vesicles and vesicle-free secretome of the protozoa Acanthamoeba castellanii under homeostasis and nutritional stress and their damaging potential to host cells
Source: Virulence. 2018 May 4;9(1):818–36. doi: 10.1080/21505594.2018.1451184 (PMC5955443; doi:10.1080/21505594.2018.1451184)

## Slide 1
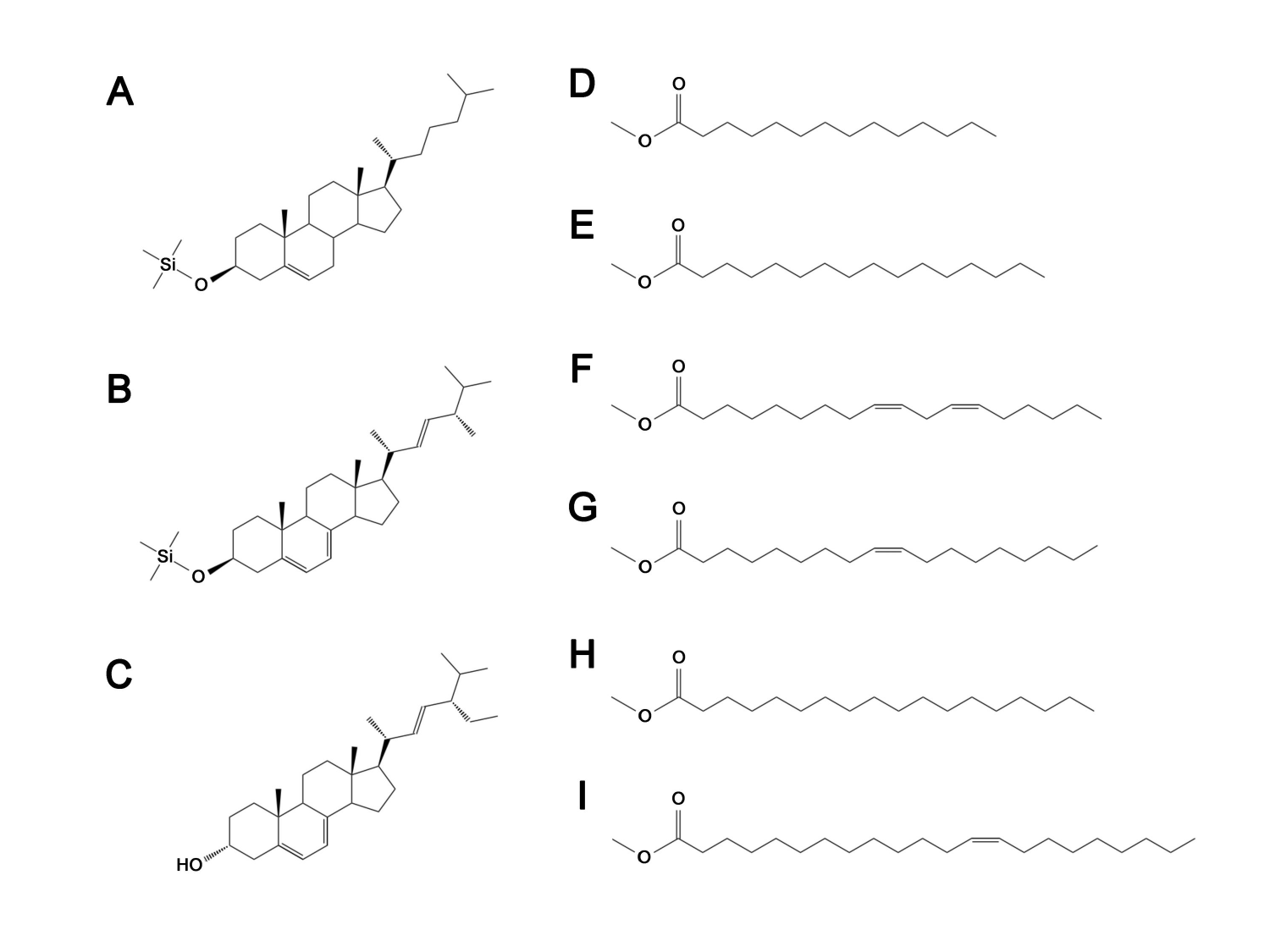

## Slide 2
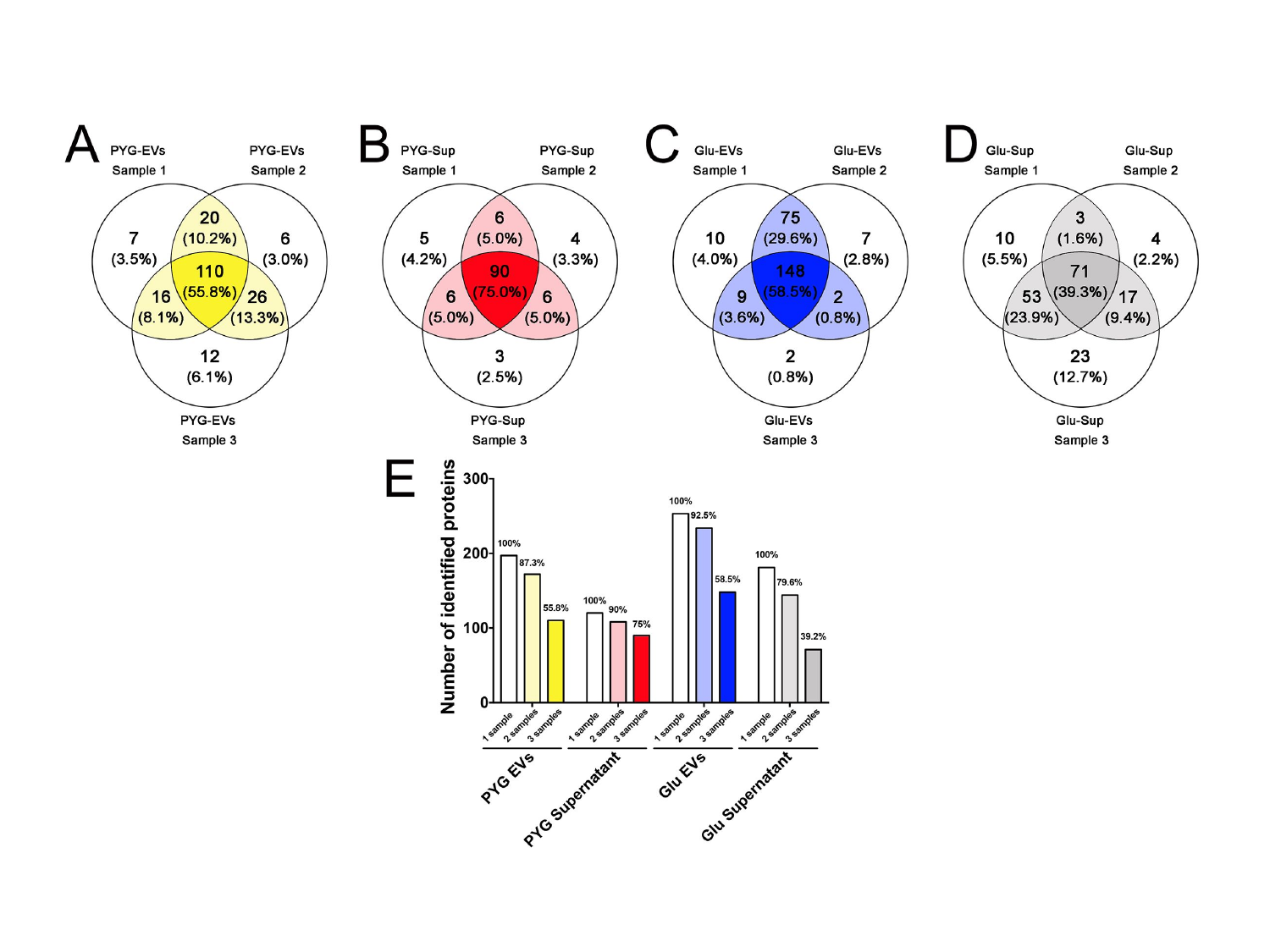

## Slide 3
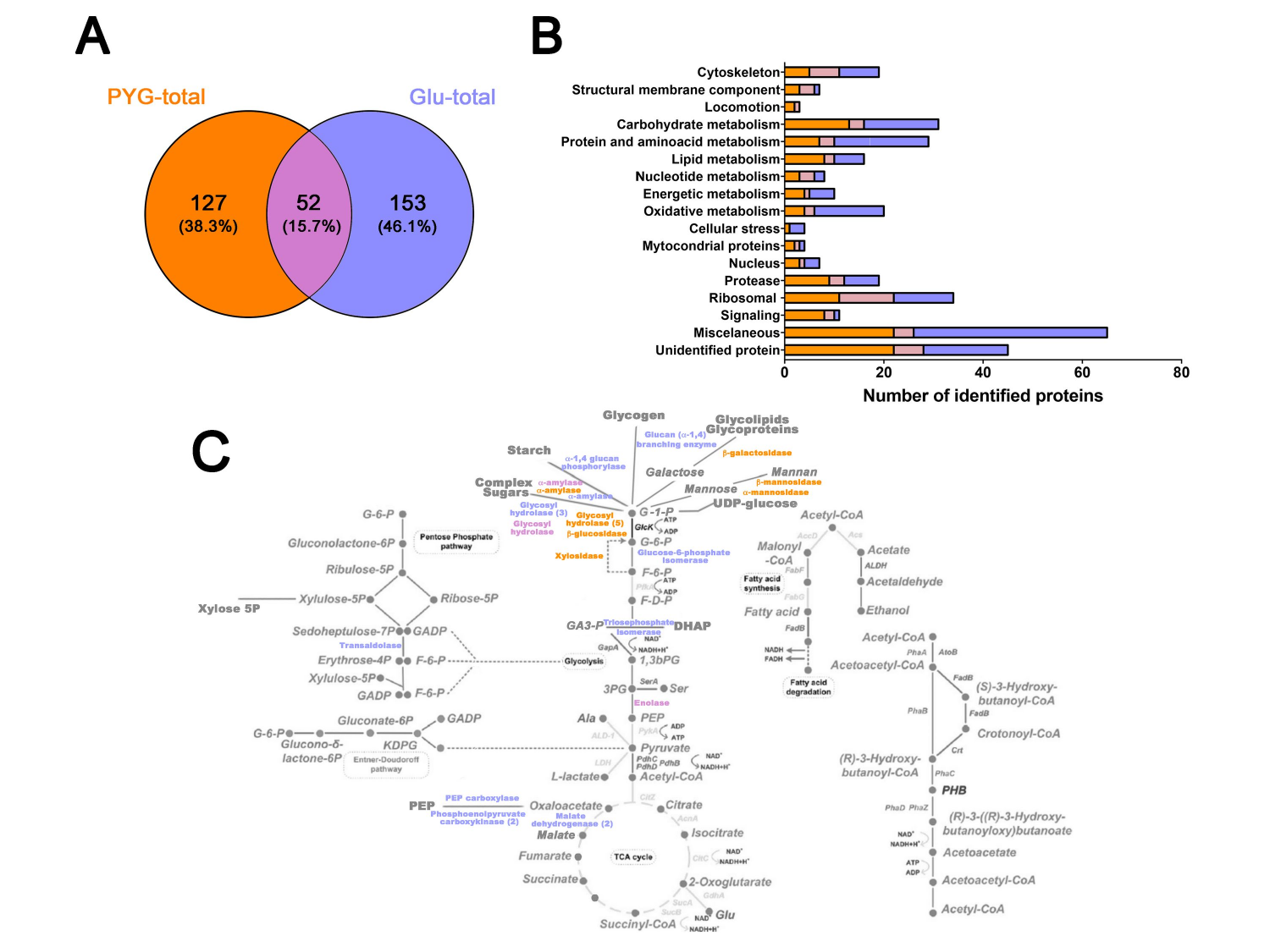

## Slide 4
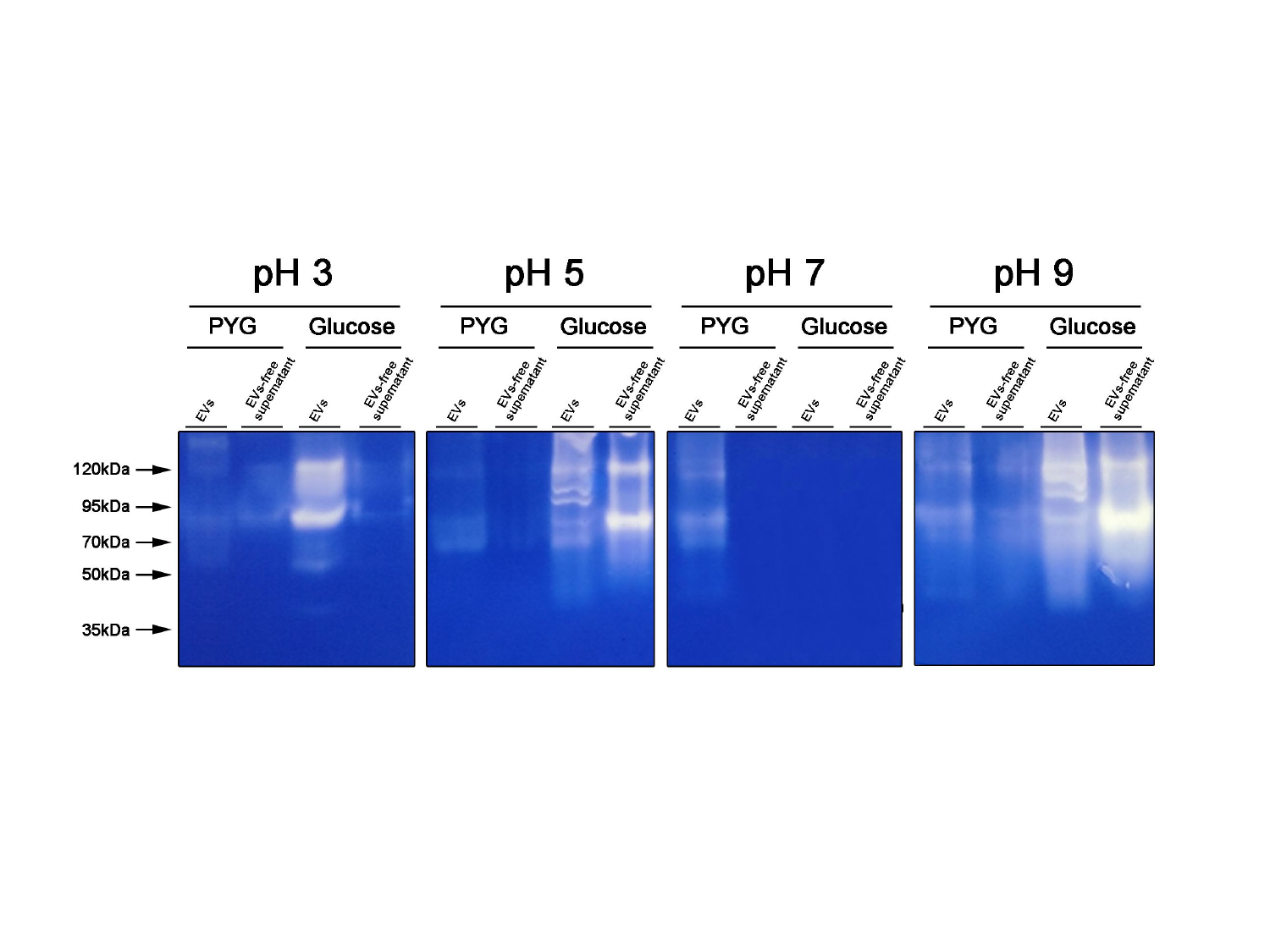

Supplement: 1451184.zip [file kvir-09-01-1451184-s001.zip › 1451184/Supplementary Figures Virulence Ac EVs_submitted.pptx]
